# Machine Learning Used in Communicable Disease Control: A Scoping Review

**DOI:** 10.3389/phrs.2026.1608074

**Published:** 2026-02-13

**Authors:** Sharon Birdi, Atushi Patel, Roxana Rabet, Navreet Singh, Steve Durant, Tina Vosoughi, Faris Kapra, Mahek Shergill, Elnathan Mesfin, Carolyn Ziegler, Shehzad Ali, David Buckeridge, Marzyeh Ghassemi, Jennifer Gibson, Ava John-Baptiste, Jillian Macklin, Melissa Mccradden, Kwame Mckenzie, Sharmistha Mishra, Parisa Naraei, Akwasi Owusu-Bempah, Laura Rosella, James Shaw, Ross Upshur, Andrew D. Pinto

**Affiliations:** 1 Upstream Lab, MAP Centre for Urban Health Solutions, Li Ka Shing Knowledge Institute, St. Michael’s Hospital, Unity Health Toronto, Toronto, ON, Canada; 2 Michael G. DeGroote School of Medicine, Faculty of Health Sciences, McMaster University, Hamilton, ON, Canada; 3 Library Services, Unity Health Toronto, Toronto, ON, Canada; 4 Department of Epidemiology and Biostatistics, Schulich School of Medicine and Dentistry, Western University, London, ON, Canada; 5 Department of Health Sciences, Faculty of Sciences, University of York, Heslington, Yorkshire and the Humber, United Kingdom; 6 WHO Collaborating Centre for Knowledge Translation and Health Technology Assessment in Health Equity, Ottawa, ON, Canada; 7 Department of Epidemiology, Biostatistics and Occupational Health, School of Population and Global Health, Faculty of Medicine and Health Sciences, McGill University, Montreal, QC, Canada; 8 Department of Electrical Engineering and Computer Science, School of Engineering, Massachusetts Institute of Technology, Cambridge, MA, United States; 9 Institute for Medical Engineering and Science, School of Engineering, Massachusetts Institute of Technology, Cambridge, MD, United States; 10 Joint Centre for Bioethics, University of Toronto, Toronto, ON, Canada; 11 Department of Anesthesia and Perioperative Medicine, Schulich School of Medicine and Dentistry, Western University, London, ON, Canada; 12 Schulich Interfaculty Program in Public Health, Schulich School of Medicine and Dentistry, Western University, London, ON, Canada; 13 Undergraduate Medical Education, Faculty of Medicine, University of Toronto, Toronto, ON, Canada; 14 Department of Bioethics, The Hospital for Sick Children, Toronto, ON, Canada; 15 Genetics and Genome Biology, SickKids Research Institute, Toronto, ON, Canada; 16 Division of Clinical Public Health, Dalla Lana School of Public Health, University of Toronto, Toronto, ON, Canada; 17 Wellesley Institute, Toronto, ON, Canada; 18 The Centre for Addiction and Mental Health, Toronto, ON, Canada; 19 Division of Infectious Diseases, Department of Medicine, University of Toronto, Toronto, ON, Canada; 20 MAP Centre for Urban Health Solutions, Li Ka Shing Knowledge Institute, St. Michael’s Hospital, Unity Health Toronto, Toronto, ON, Canada; 21 Institute of Medical Science, Temerty Faculty of Medicine, University of Toronto, Toronto, ON, Canada; 22 Institute of Health Policy Management and Evaluation, Dalla Lana School of Public Health, University of Toronto, Toronto, ON, Canada; 23 Department of Epidemiology, Dalla Lana School of Public Health, University of Toronto, Toronto, ON, Canada; 24 Institute for Clinical Evaluative Sciences, Toronto, ON, Canada; 25 Department of Computer Science, Toronto Metropolitan University, Toronto, ON, Canada; 26 Department of Sociology, Faculty of Arts and Science, University of Toronto, Toronto, ON, Canada; 27 Institute for Better Health, Trillium Health Partners, Toronto, ON, Canada; 28 Department of Physical Therapy, Faculty of Medicine, University of Toronto, Toronto, ON, Canada; 29 Department of Family and Community Medicine, Faculty of Medicine, University of Toronto, Toronto, ON, Canada; 30 Department of Family and Community Medicine, St. Michael’s Hospital, Toronto, ON, Canada; 31 Temerty Faculty of Medicine, University of Toronto, Toronto, ON, Canada

**Keywords:** public health, communicable diseases, machine learning, artificial intelligence, population health, scoping review

## Abstract

**Objectives:**

Communicable diseases continue to threaten global health, with COVID-19 as a recent example. Rapid data analysis using machine learning (ML) is crucial for detecting and controlling outbreaks. We aimed to identify how ML approaches have been applied to achieve public health objectives in communicable disease control and to explore algorithmic biases in model design, training, and implementation, and strategies to mitigate these biases.

**Methods:**

We searched MEDLINE, Embase, Cochrane Central, Scopus, ACM DL, INSPEC, and Web of Science to identify peer-reviewed studies from 1 January 2000, to 15 July 2022. Included studies applied ML models in population and public health to address ten communicable diseases with high prevalence.

**Results:**

28,378 citations were retrieved, and 209 met our inclusion criteria. ML for communicable diseases has risen since 2020, particularly for SARS-CoV-2 (n = 177), followed by malaria, HIV, and tuberculosis. Eighteen studies (8.61%) considered bias, and only eleven implemented mitigation strategies.

**Conclusion:**

A growing number of studies used ML for disease surveillance. Addressing biases in model design should be prioritized in future research to improve reliability and equity in public health outcomes.

## Introduction

Communicable diseases, caused by pathogenic microorganisms such as viruses, bacteria, parasites, or fungi, remain a significant global public health threat [1]. Despite advances in medicine and sanitation, communicable diseases account for a substantial share of the global disease burden [2]. According to the World Health Organization (WHO), communicable diseases, including lower respiratory infections, diarrheal diseases, and tuberculosis were responsible for 8 of the top 10 causes of death in low-income countries in 2021 [3]. The COVID-19 pandemic further underscored the health, economic, and social impacts of emerging pathogens.

Machine learning (ML) has the potential to transform communicable disease management by enabling early detection and prediction of outbreaks and pandemics [4, 5]. In healthcare, ML is increasingly used to process and identify patterns in large amounts of data from electronic health records and wearable devices [4]. In public health, ML algorithms can analyze complex interactions in data from multiple sources to support more accurate predictions of emerging health threats, to define the scale of an outbreak, and to rapidly evaluate communicable disease control interventions [6, 7]. These models have seen wide application during the COVID-19 pandemic, where they were used to forecast trends, support clinical decisions, and guide resource allocation [6, 8]. [6, 8, 9] However, the extent of use of ML in population and public health remains unclear, highlighting the need for a comprehensive review of recent approaches in this field.

The objective of this study was to conduct a scoping review to identify studies that use ML to address population and public health challenges related to communicable diseases. Themes explored included whether and how teams considered bias during the design, training, and implementation of ML models. Given the well-documented risks of bias in the development and implementation of ML models for public health, we prioritized this aspect to underscore the importance of fairness and equity in model outcomes.

## Methods

This scoping review followed the Arksey and O’Malley guidelines for scoping reviews [10, 11] and the Preferred Reporting Items for Systematic Reviews and Meta-Analyses Extension for Scoping Reviews (PRISMA-ScR) guidelines [12]. Our protocol was published by the Open Science Framework (https://osf.io/xydut/).

### Search Strategy

An experienced information specialist (CZ) helped develop and conduct a comprehensive search of the peer-reviewed, indexed literature. The following databases were searched from 1 January 2000, to 15 July 2022: Medline (Ovid), Embase (Ovid), Cochrane Central Register of Controlled Trials and Cochrane Database of Systematic Reviews (Ovid), Scopus, ACM Digital Library, INSPEC, and Web of Science’s Science Citation Index, Social Sciences Citation Index, and Emerging Sources Citation Index. The publication date ranged from 2000 to 2022 was selected to capture ML models that leverage modern computing techniques and recent data advancements. The search used a combination of subject headings and keywords, adapted for each database, for the broad concepts of artificial intelligence combined with the following communicable diseases: lower respiratory infections, diarrheal diseases, tuberculosis, HIV, malaria, meningitis, measles, pertussis (whooping cough), hepatitis, SARS-CoV-2. All languages were included in the search (Supplementary Material S2). We limited our search to these 10 specific communicable diseases based on their high global prevalence and public health impact [3]. These diseases were selected to provide a focused analysis while ensuring relevance to current population health priorities.

### Eligibility Criteria

To be eligible, studies had to meet the following criteria during both title/abstract and full-text screening: (1) focus on population-level implications or adopt a public health approach; (2) address at least one of the following conditions: lower respiratory infections, diarrheal diseases, tuberculosis, HIV, malaria, meningitis, measles, pertussis (whooping cough), hepatitis, or SARS-CoV-2; (3) utilize at least one ML model to tackle a real-world population or public health challenge. There were no language restrictions, and all study designs, except for review articles, were considered.

Studies were excluded if: (1) they did not have population-wide implications or a public health approach; (2) they did not focus on any of the conditions listed in the inclusion criteria or focused only on complications and related conditions; (3) no real-world data was used; or (4) they were commentaries, letters, editorials, conference proceedings, or dissertations.

### Study Selection and Data Collection Process

Citations from all databases were imported into DistillerSR [13] for the initial title and abstract review. Each citation was reviewed independently by two reviewers (RR, TV, AP, EM, NS) using the eligibility criteria to determine inclusion or exclusion for full-text review. Any conflicts during this process were solved through discussion with a third author (SB). Full articles were retrieved for further eligibility screening, and studies that met the eligibility criteria were included. The final set of studies included in this scoping review includes only those that passed the full-text screening process. Five members of the study team assisted with data extraction (RR, TV, AP, EM, NS).

The following data were extracted: author(s), title, journal, publication year, ML application type(s), the intended purpose of ML, study design, intervention (if applicable), results, jurisdiction, data sources, unit(s) of analysis, sample size, demographics, identification of any potential algorithmic bias in the ML model (biases related to gender, sex, ethnicity, socioeconomic status), transferability to low- and middle-income countries, bias mitigation strategies, CDs targeted, target population and setting, intended users, and impact reported by the author. We also noted if information was unavailable from an article or if any additional sources of algorithmic bias (e.g., age-related bias) were discussed.

### Data Synthesis

We used a narrative synthesis to review and summarize the objectives, ML algorithms, and relevance of each study. We focused on how these studies used ML to characterize and detect communicable disease cases and outbreaks, detailing the application and implications of using ML algorithms on specific communicable diseases. We organized the studies by the communicable disease explored and identified common limitations found in the studies, such as small data sets and generalizability issues.

## Results

### Study Selection and Characteristics

**FIGURE 1:**
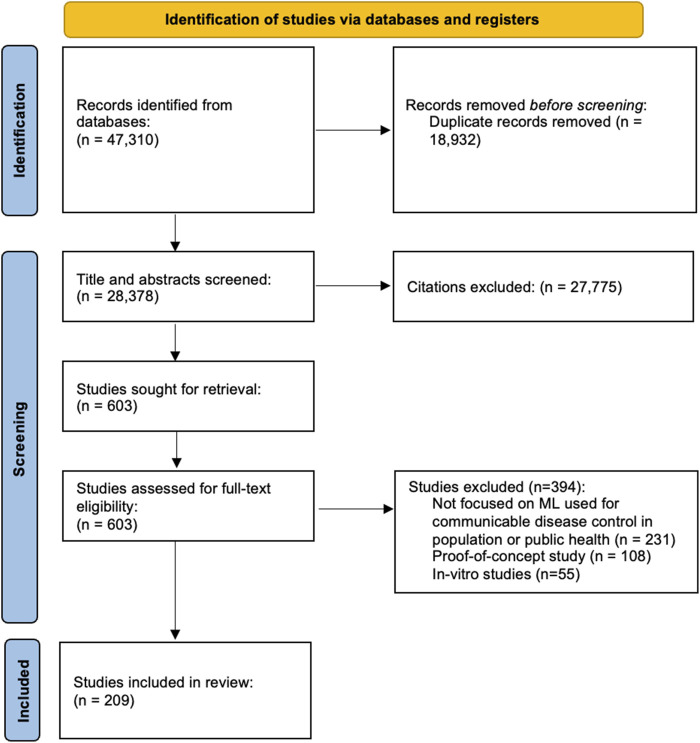
Selection process of eligible studies from all identified citations (Toronto, Canada, 2026).

Our initial search identified 47,310 citations. After removing 18,932 duplicates, 28,378 citations were double-screened. Following title and abstract screening, 603 studies were included for full-text review. Following full-text screening, 394 of these studies were excluded, leaving 209 studies that met our criteria for this review (Figure 1).

The number of studies using ML in communicable disease control at the population level or for public health purposes has increased over time. The first study was published in 2005, and only 10 (4.8%) studies were published between 2000–2015, and most studies (n = 199, 95.2%) were published between 2020 and 2023. A large number of studies were conducted by teams in the USA (n = 31, 14.8%), India (n = 18, 8.6%) or China (n = 15, 7.2%), but ML approaches are now common around the world (Table 1).

**TABLE 1:** Distribution of studies by country where research on machine learning in population or public health occurred (Toronto, Canada, 2026).

Country	Frequency	Percentage (%)
Algeria	1	0.48
Bangladesh	5	2.39
Brazil	1	0.48
Burkina Faso	1	0.48
Burundi	1	0.48
Canada	3	1.44
China	15	7.18
Colombia	1	0.48
Egypt	1	0.48
Eswatini	1	0.48
France	1	0.48
Germany	3	1.44
India	18	8.61
Indonesia	3	1.44
Iran	8	3.83
Iraq	3	1.44
Israel	1	0.48
Italy	2	0.96
Japan	1	0.48
Jordon	1	0.48
Kuwait	1	0.48
Malaysia	1	0.48
Mexico	5	2.39
Nigeria	2	0.96
Pakistan	4	1.91
Peru	1	0.48
Philippines	1	0.48
Portugal	1	0.48
Qatar	1	0.48
Romania	1	0.48
Saudi Arabia	8	3.83
Serbia	1	0.48
Somalia	1	0.48
South Africa	2	0.96
South Korea	3	1.44
Spain	3	1.44
Taiwan	1	0.48
Tanzania	1	0.48
Thailand	1	0.48
Turkey	3	1.44
U.S.A.	31	14.83
U.K.	2	0.96
Ukraine	1	0.48
Zambia	1	0.48

### Application Aims

Of the included studies, 9.57% (n = 20) [14–33] compared various ML models/approaches, 35.9% (n = 75) [34–51], [52–71], [72–91], [92–108] modelled population-level disease incidence as the outcome, 4.78% (n = 11) [109–119] modelled population-level disease risk, 7.18% (n = 15) [120–134] focused on disease surveillance, specifically identifying cases, 1.91% (n = 4) [135–138] evaluated the effectiveness of a public health intervention on disease incidence, and 40.2% (n = 84) [139–151], [152–171], [172–190], [191–205], [206–221] of studies were identified as having multiple application aims.

### Data Sources

Most studies sourced data from biomedical databases including aggregates of research-based data, such as clinical trials or populations health studies (n = 160, 76.6%) [14–20], [24, 25, 27], [30–32], [34–52], [55–60], [62–68], [70–82], [84–89], [91, 92, 95], [98–106], [110–117], [119], [123–125], [128, 130], [135–138], [140–148], [150, 151, 153, 154, 157, 158, 160, 161, 163], [165–168], [172, 173], [175–177], [179–196], [198, 200–207], [209, 211–215], [217–219, 221], followed by longitudinal databases (n = 24, 11.48%) [23, 28, 29, 33, 53, 61, 83, 93, 108, 118, 129, 139, 162], textual elements drawn from social media (n = 10, 4.78%) [120–122, 126, 127, 131, 134, 149, 174, 197], electronic medical records (n = 2, 0.96%), and other data sources (i.e., Google Search Trends, Meteorological and Environmental data) (n = 2, 0.96%) [199, 220]. A combination of data sources was utilized in 11 (5.26%) [54, 69, 94, 96, 97, 132, 133, 164, 208, 210, 216] of the included studies.

### Communicable Diseases

A majority of studies (n = 177, 84.7%) [14–16], [18–27], [30–32], [35–45], [49–57, 59], [62–74], [76–107], [109–117], [119, 121, 124, 125], [127–131], [133, 134], [136–156], [158–166], [168, 169], [171–178], [182–188], [191–192], [194–197], [200–218, 221] focused on SARS-CoV-2. The most commonly studied communicable diseases after SARS-CoV-2 were malaria (n = 9, 4.31%) [17, 48, 58, 60, 61, 75, 171, 219, 220], HIV (n = 8, 3.83%) [28, 29, 120, 122, 123, 135, 190, 193], tuberculosis (n = 5, 2.39%) [33, 34, 132, 189, 199], diarrheal diseases (n = 4, 1.91%) [46, 47, 179, 180], hepatitis (n = 3, 1.44%) [118, 157, 198], and measles (n = 1, 0.48%) [167]. Multiple communicable diseases were the focus of two (0.96%) [126, 181] studies included in the sample.

### Technical Approaches

A variety of specialized algorithms/models were employed across studies, such as ARIMA (AutoRegressive Integrated Moving Average) and ANFIS (Adaptive Neuro Fuzzy Interference System) (n = 127, 60.8%) [2–6], [15, 17, 21, 23, 25, 26, 28, 29], [31–38], [40, 42, 44, 45], [48–53], [57–59], [62, 63, 65, 67, 69, 71, 72, 74], [76–80], [82, 84], [86–90], [92, 94, 95, 97], [100–103], [106–114], [117, 118, 121], [124–126], [128, 130, 133, 134, 139, 141, 142, 149, 151, 153, 154], [159–161], [163, 165], [169–171], [173–179], [181–183], [185–188], [190, 191–197], [200, 201, 208]. Mixed technical approaches (e.g., combination of natural language processing, and neural networks) were employed in approximately 1 of 3 studies (n = 61, 29.2%) [20–24], [26, 30–32], [36, 39, 42, 49, 53, 56, 59, 66], [73, 76, 80, 82, 93, 97], [110, 128, 131, 132, 134, 135, 141], [143, 144, 147, 150], [152, 155–160, 162], [167–170, 174, 179], [180, 184, 191, 195, 200, 202, 210, 211], [214–218]. Supervised learning algorithms were employed in 12 studies (n = 5.74%) [51, 58, 68, 72, 87, 95, 103, 111, 116, 127, 148, 219], and deep learning neural networks were employed in 9 studies (4.31%) [24, 67, 78, 85, 105, 117, 149, 164, 176].

### Consideration of Bias and Its Mitigation

A total of 18 studies (8.61%) [68, 71, 94, 95, 101], [105–107], [117, 126, 133, 152, 153, 159, 178, 186, 193, 194] of 209 explicitly considered bias. Of the 18, five studies [80, 107, 197, 205, 206] considered demographic bias stemming from age, sex, or ethnicity which reflected a lack of representation of certain groups or the exclusion of data on specific populations. Four studies [83, 119, 171, 190] considered bias stemming from socioeconomic status which arose from limited data or underrepresentation of lower socioeconomic groups. Two studies [117, 118] did not specify the specific type of bias, and seven studies [106, 113, 129, 138, 145, 164, 165] indicated considering bias stemming from other sources, such as measurement and statistical biases. In addition, of the studies that did consider bias, 11 studies [94, 95, 105–107, 126, 133, 152, 159, 194] implemented a bias mitigation strategy to address these concerns.

## Discussion

This scoping review identified 209 studies that applied ML models in population and public health to address communicable diseases. Most studies focused on SARS-CoV-2, with modelling disease incidence being the most common application.

The COVID-19 pandemic drove a rapid growth in ML research aimed at predicting case trends and guiding public health interventions. Studies applied a range of models, from traditional regression to deep learning, to predict case trends and inform interventions. For example, Devaraj et al. used deep learning to forecast SARS-CoV-2 cases, highlighting the model’s ability to learn temporal dependencies and trends [211]. Castillo-Olea et al. compared logistic regression and neural networks to identify early-stage SARS-CoV-2 cases in a hospital setting [109]. Both ML models were successful in evaluating differing variables, effectively identifying early-stage cases of SARS-CoV-2 [109]. Nguyen et al. examined BeCaked, a novel model combining the Susceptible-Infectious-Recovered-Deceased (SIR-D) compartmental model and the Variational Autoencoder (VAE) neural network, to forecast SARS-CoV-2 cases [153]. BeCaked aimed to overcome the limitations of the individual ML models to ensure effectiveness and provide reliable predictions of SARS-CoV-2 cases [153]. Overall, our analysis found that studies frequently relied on specialized or hybrid models to address the shortcomings of standalone approaches.

Specialized and ensemble approaches were frequently used to improve predictive performance and overcome model limitations. Ahmad et al. explored optimal models to predict SARS-CoV-2 cases, by comparing ML and DL models such as linear regression, support vector regression, and long short-term memory (LSTM) [14]. Lucas et al. approached SARS-CoV-2 forecasting by using a modified LSTM system, COVID-LSTM, which integrates spatiotemporal features into an LSTM model [171]. Likewise, Arik et al. extended the Susceptible-Exposed-Infectious-Removed (SEIR) model by proposing an AI-augmented epidemiology framework for SARS-CoV-2 forecasting [207] These efforts underscore the importance of accurate forecasting tools to inform outbreak response and public health planning.

Forecasting remains a critical application of ML, particularly in pandemic response. Many studies turned to novel approaches to explore the prediction accuracy of models. Ghazaly et al. examined prediction accuracy for SARS-CoV-2 cases using a Non-linear Auto-Regressive Network (NAR) network [44]. This method is similar to ANN, except that it depends on past information for future forecasting. Accurate predictions of SARS-CoV-2 spread are critical for health systems globally as they facilitate preventative measures and timely interventions, helping to manage risks and demands [222]. The COVID-19 pandemic has put immense pressure on healthcare systems worldwide, highlighting the need for reliable and accurate forecasting models [223].

Studies also addressed malaria, HIV, tuberculosis, and diarrheal diseases. These models often incorporated meteorological or demographic data to improve predictive accuracy. Abdukar et al. [46] used ANN to forecast the incidence of diarrheal diseases in Nigeria, while Fang et al. [179] applied an RF model to predict infectious diarrhea in China. Brown et al. developed a predictive ML system using generalized linear models (GLM), ensemble methods, and SVM for malaria estimation [61]. Similarly, Mfisimana et al. used GLM and ANN to predict malaria cases. Given the complexity of malaria and its interventions, multivariate models are preferred, as no single intervention can fully eliminate the disease [75]. Non-linear models were frequently applied to HIV and tuberculosis to account for complex and dynamic transmission patterns [29, 132, 135]. These included backpropagation neural networks, convolutional neural networks, and ARIMA models.

A central objective of this review was to assess how studies addressed bias. Some models incorporated strategies to mitigate algorithmic bias. A study by Almazroi & Usmani used Tree-based ensemble methods in their model design, such as RFs or XGBoost, to reduce bias caused by combining various predictor models into a single model [184]. Maria-Gomez addressed bias in model implementation by adjusting models for age or sex [107]. Price et al identified bias in model training, noting that rural areas and infection incidence were not accurately represented in training datasets [164].

Applying ML models in public and population health to detect or characterize communicable diseases requires careful attention to data quality. The performance and reliability of these models depend on the consistency, completeness, and accuracy of the data used for training. Many studies reported challenges such as missing, inconsistent, inaccurate, or duplicate data, which can significantly reduce the predictive accuracy and generalizability of ML models [223].

Interpretability was another underexamined area. While ML models can support public health decision-making, opaque algorithms may limit their utility in practice. Transparent models and explainable outputs are essential to ensure accountability, particularly when predictions affect resource allocation, outbreak response, or population health planning [224].

This review has several strengths. First, it involves a comprehensive search across multiple databases, the use of clearly defined inclusion and exclusion criteria and the double-review screening process. This review also has limitations. Despite a broad search strategy designed to capture all subtypes of ML applications in public and population health to address communicable diseases, some relevant articles may have been inadvertently excluded due to our global scope and the inherent limitations of indexing. Additionally, grey literature was excluded from the search.

## Conclusion

This scoping review highlights the potential of ML applications in public and population health for predicting and characterizing communicable diseases. Although this study examined a broad spectrum of studies on the development, implementation, and comparison of these models, it’s clear that using ML for communicable diseases in public health is still an evolving field, with ongoing challenges remaining. There is a need for more representative datasets for training models and more rigorous validation to ensure reliable, accurate, and acceptable tools. Future research should focus further on identifying and addressing biases that can emerge during the design, training, and implementation of ML models used in public and population health.
