# Supplementary material for: Machine Learning Used in Communicable Disease Control: A Scoping Review
Source: Public Health Rev. 2026 Feb 13;47:1608074. doi: 10.3389/phrs.2026.1608074 (PMC12945845; doi:10.3389/phrs.2026.1608074)
Supplement: Supplementary file 1 [file Table1.docx]

**Table 1. Summary of characteristics of included studies**

| **Author (year)** | **Title** | **Country** | **Data Source** | **Communicable Disease(s)** | **ML Application Type(s)** | **Intended Purpose of ML** | **Was bias identified during model design, training or implementation** | **Type of ML Bias Identified** | **How was ML bias mitigated?** |
| --- | --- | --- | --- | --- | --- | --- | --- | --- | --- |
| Rauf (2023) | Time series forecasting of COVID-19 transmission in Asia Pacific countries using deep neural networks | Pakistan | Biomedical Databases | SARS-CoV-2 | LSTM, RNN, and Gated Recurrent Units (GRU) | Modelling disease incidence in population AND comparison of models/approaches | N/A | N/A | N/A |
| Lucas (2023) | A spatiotemporalmachine learning approach to forecasting COVID-19 incidence at the county level in the USA | USA | Longitudinal Survey Data | SARS-CoV-2 | COVID-LSTM, CMU-TimeSeries, UMass-MechBayes, Google_Harvard-CPF, FAIR-NRAR, COVIDhub-baseline, COVIDhub-ensemble | Comparison of models/approaches; modelling disease incidence in population | Model Implementation | SES, ethnicity | defined a hybrid LSTM model to incorporate variables alongside existing ones to see if the variables would imrpove forecasts our existing temporal ones |
| Renukadevi (2023) | Covid-19 Forecasting with Deep Learning-based Half-binomial Distribution Cat Swarm Optimization | India | Biomedical Databases | SARS-CoV-2 | CNN | Modelling disease incidence in population | N/A | N/A | N/A |
| Raheja (2023) | Machine learning-based diffusion model for prediction of coronavirus-19 outbreak | India | Biomedical Databases | SARS-CoV-2 | SVM, Logistic regression and CNN | Modelling disease incidence in population AND comparison of models/approaches | N/A | N/A | N/A |
| Grekousis (2022) | Ranking the importance of demographic, socioeconomic, and underlying health factors on US COVID-19 deaths: A geographical random forest approach | USA | Biomedical Databases | SARS-CoV-2 | Geographical Random Forest (GRF) | Modelling disease incidence in population | N/A | N/A | N/A |
| Jiang (2022) | COVID-19 Surveiller: toward a robust and effective pandemic surveillance system basedon social media mining | USA | Social Media | SARS-CoV-2 | RIMA, Prophet, LSTM, Message passing neural network-based models (MPNN) | Surveillance | N/A | N/A | N/A |
| Jiang (2022) | Artificial neural network-based estimation of COVID-19 case numbers and effective reproduction rate using wastewater-based epidemiology | USA | Biomedical Databases AND Environmental datasets | SARS-CoV-2 | ANN | Modelling disease incidence in population | N/A | N/A | N/A |
| Jantzen (2022) | Socio-Demographic Factors Associated With COVID-19 Vaccine Hesitancy Among Middle-Aged Adults During the Quebec's Vaccination Campaign | Canada | Longitudinal Survey Data | SARS-CoV-2 | Hybrid tree-based model | Surveillance | Model Training | Selection bias (non-respondents could have different opinions about vaccination compared to respondents) | N/A |
| Hussein (2022) | Short-Term and Long-Term COVID-19 Pandemic Forecasting Revisited with the Emergence of OMICRON Variant in Jordan | Jordan | Biomedical Databases | SARS-CoV-2 | Short-term forecast (STF) model; Long-term forecast (LTF) model; Hybrid forecast (HF) model | Modelling disease incidence in population | N/A | N/A | N/A |
| Haq (2022) | Prediction of COVID-19 Pandemic in Bangladesh: Dual Application of Susceptible Infective-Recovered (SIR) and Machine Learning Approach | Bangladesh | Biomedical Databases | SARS-CoV-2 | PROPHET Forecasting Procedure | Modelling disease incidence in population | Model Training | N/A | N/A |
| Han (2022) | Impact analysis of environmental and social factors on early-stage COVID-19 transmission in China by machine learning | China | Biomedical Databases | SARS-CoV-2 | Gradient Boosting Decision Tree, RF, Elastic Net, 2-Layer ANN | Surveillance | Model Implementation | N/A | N/A |
| Gupta (2022) | Predicting and monitoring COVID-19 epidemic trends in India using sequence-to-sequence model and an adaptive SEIR model | India | Biomedical Databases | SARS-CoV-2 | Adapting SEIR model | Modelling disease incidence in population | N/A | N/A | N/A |
| Malinzi (2022) | Determining COVID-19 Dynamics Using Physics Informed Neural Networks | Eswatini | Longitudinal Survey Data | SARS-CoV-2 | Physics Informed Neural Network structure (PINN) | Modelling disease incidence in population | N/A | N/A | N/A |
| Kalezhi (2022) | Modelling Covid-19 infections in Zambia using data mining techniques | Zambia | Biomedical Databases | SARS-CoV-2 | J48 decision tree, Multilayer Perceptron, Naïve Bayes, Random Forest, Support Vector Machine, K Nearest Neighbour, Logistic Regression | Modelling disease incidence in population AND comparison of models/approaches | N/A | N/A | N/A |
| Gollapalli (2022) | Ensemble Machine Learning Model to Predict the Waterborne Syndrome | Bangladesh | Biomedical Databases | Diarrheal Diseases | Ensemble Model | Modelling disease incidence in population | N/A | N/A | N/A |
| Galasso (2022) | A random forest model for forecasting regional COVID-19 cases utilizing reproduction number estimates and demographic data | USA | Biomedical Databases | SARS-CoV-2 | RF | Modelling disease incidence in population | N/A | N/A | N/A |
| Fritz (2022) | Combining graph neural networks and spatio-temporal disease models to improve the prediction of weekly COVID-19 cases in Germany | Germany | Biomedical Databases/Facebook | SARS-CoV-2 | Graphical Neural  Network  (GNN) | Modelling disease incidence in population | N/A | N/A | N/A |
| Favas (2022) | Country differences in transmissibility, age distribution and case-fatality of SARS-CoV-2: a global ecological analysis | Global | Biomedical Databases | SARS-CoV-2 | RF, Linear regression | Modelling risk in population | N/A | N/A | N/A |
| Nguyen (2022) | BeCaked: An Explainable Artificial Intelligence Model for COVID‑19 Forecasting | Australia, Italy, Russia, Spain, UK, USA | Longitudinal Survey Data | SARS-CoV-2 | BeCaked (combination of Susceptible-Infectious-Recovered-Deceased (SIRD) compartmental model and Variational Autoencoder (VAE)) | Comparison of models/approaches; modelling disease incidence in population | N/A | N/A | N/A |
| Lee (2022) | Symptom-Based COVID19 Screening Model Combined with Surveillance Information | Israel | Longitudinal Survey Data | SARS-CoV-2 | Logistic regression, 1D ResNet | Comparison of models/approaches | N/A | N/A | N/A |
| Chikusi (2022) | Machine Learning Model for Prediction and Visualization of HIV Index Testing in Northern Tanzania | Tanzania | Biomedical Databases | HIV/ | RF, XGBoost, ANN | Evaluating effectiveness of intervention | N/A | N/A | N/A |
| Pérez-Ortega (2022) | Application of Data Science for Cluster Analysis of COVID-19 Mortality According to Sociodemographic Factors at Municipal Level in Mexico | Mexico | Longitudinal Survey Data | SARS-CoV-2 | Batch Foundation Methodology for Data Science (FMDS) | Modelling disease incidence in population | N/A | N/A | N/A |
| Kianfar (2022) | Spatio-temporal modeling of COVID-19 prevalence and mortality using artificial neural network algorithms | Iran | Biomedical Databases and SDH data | SARS-CoV-2 | ANN | Modelling risk in population | Model Training | Response bias (it is most likely that some countries have not provided accurate statistics about COVID-19 prevalence and deaths) | No |
| Khurana (2022) | An Intelligent Fine-Tuned Forecasting Technique for Covid-19 Prediction Using Neuralprophet Model | India | Biomedical Databases | SARS-CoV-2 | RF | Modelling disease incidence in population | N/A | N/A | N/A |
| Mansour (2022) | Spatial Assessment of COVID-19 First-Wave Mortality Risk in the Global South | Global South | Longitudinal Survey Data | SARS-CoV-2 | ANN, MLP | Modelling disease incidence in population; comparison of models | N/A | N/A | N/A |
| Zhou (2022) | Interpretable Temporal Attention Network for COVID-19 forecasting | USA | Biomedical Databases | SARS-CoV-2 | ITAnet, CNN, LSTM, Transformer, and TFT | Modelling disease incidence in population | N/A | N/A | N/A |
| Zandavi (2022) | Dynamic Hybrid Model to Forecast the Spread of COVID-19 Using LSTM and Behavioral Models Under Uncertainty | USA, Brazil, India, Russia, Great Britain, France, Italy, Spain, Turkey, Germany | Biomedical Databases | SARS-CoV-2 | RNN, LSTM, Hybrid Model | Comparison of models/approaches | N/A | N/A | N/A |
| Xu (2022) | Foredcasting COVID-19 new cases using deep learning methods | Brazil, India, and Russia | Biomedical Databases | SARS-CoV-2 | LSTM, CNN, CNN-LSTM | Modelling disease incidence in population | N/A | N/A | N/A |
| Lucas (2022) | Improving disaggregation models of malaria incidence by ensembling non-linear models of prevalence | Madagascar, Colombia, Indonesia and Senegal | Longitudinal Survey Data | Malaria | RF, GBM, ENET, NNET, PPR | Comparison of models/approaches; modelling disease incidence in population | N/A | N/A | N/A |
| Tajmouati (2022) | Modeling COVID-19 Confirmed Cases Using a Hybrid Model | Pakistan, Tunisia, Indonesia, Malaysia, India and South Korea | Biomedical Databases | SARS-CoV-2 | Autoregressive Integrated Moving Average + Nonlinear Autoregression Neural Network (ARIMA-NARNN), ARIMA, NNAR, and ARIMA-WBF | Modelling disease incidence in population | N/A | N/A | N/A |
| Sultana (2022) | COVID-19 Pandemic Prediction and Forecasting Using Machine Learning Classifiers | Asian countries (unspecified) | Biomedical Databases | SARS-CoV-2 | Linear regression, MLP, and Vector Auto Regression | Modelling disease incidence in population | N/A | N/A | N/A |
| Silva (2022) | Predictive Analysis of COVID-19 Symptoms in Social Networks through Machine Learning | Global | Social Media | SARS-CoV-2 | KNN, Naive Bayes, DT, RF, SVM, simple Multilayer Perceptron, Convolutional Neural Networks and BERT | Surveillance/ Comparison of models/approaches | N/A | N/A | N/A |
| Rahman (2022) | The prediction of coronavirus disease 2019 outbreak on Bangladesh perspective using machine learning: a comparative study | Bangladesh | Biomedical Databases | SARS-CoV-2 | Multiple linear regression, ridge regression, lasso regression | Modelling disease incidence in population AND comparison of models/approaches | N/A | N/A | N/A |
| Caruso (2022) | Early prediction of SARS-CoV-2 reproductive number from environmental, atmospheric and mobility data: A supervised machine learning approach | Italy; Lombardy Region composed of 12 provinces | Biomedical Databases | SARS-CoV-2 | Linear regression | Modelling disease incidence in population | N/A | N/A | N/A |
| Quilodrán-Casas (2022) | Digital twins based on bidirectional LSTM and GAN for modelling the COVID-19 pandemic | UK | Biomedical Databases | SARS-CoV-2 | Generative Adversial Networks and LSTM | Modelling disease incidence in population AND comparison of models/approaches | N/A | N/A | N/A |
| Purwandari (2022) | Forecasting model of COVID-19 pandemic in Malaysia: An application of time series approach using neural network | Malaysia | Biomedical Databases | SARS-CoV-2 | Neural network: Neural Network Auto-Regressive, MLP, and Extreme Learning Machine | Modelling disease incidence in population | N/A | N/A | N/A |
| Niraula (2022) | A Bayesian machine learning approach for spatio-temporal prediction of COVID-19 cases | Spain | Longitudinal Survey Data | SARS-CoV-2 | LSTM-INLA, LSTM | Comparison of models/approaches; modelling disease incidence in population | N/A | N/A | N/A |
| Ben Yahia (2022) | Integrating Models and Fusing Data in a Deep Ensemble Learning Method for Predicting Epidemic Diseases Outbreak | China, Tunisia | Longitudinal Survey Data | SARS-CoV-2 | LSTM, CNN, DNN | Comparison of models/approaches | N/A | N/A | N/A |
| Mohamed (2022) | Evaluation of prediction models for the malaria incidence in Marodijeh Region, Somaliland | Somalia | Biomedical Databases | Malaria | SARIMA, Holt-Winters’ Exponential Smoothing, Harmonic Model, Seasonal and Trend Decomposition using Loess (STL) and ANN | Comparison of models/approaches | N/A | N/A | N/A |
| Mfisimana (2022) | Exploring predictive frameworks for malaria in Burundi | Burundi | Biomedical Databases | Malaria | GLM, ANN | Modelling disease incidence in population | N/A | N/A | N/A |
| Krivorotko (2022) | Agent-based modeling of COVID-19 outbreaks for New York state and UK: Parameter identification algorithm | USA and UK | Biomedical Databases | SARS-CoV-2 | SARIMA | Modelling disease incidence in population AND Modelling risk in population | N/A | N/A | N/A |
| Chandra (2022) | Deep learning via LSTM models for COVID-19 infection forecasting in India | India | Biomedical Databases | SARS-CoV-2 | Variations of long short term machine learning models | Modelling disease incidence in population | N/A | N/A | N/A |
| Zrieq (2022) | Analysis and modeling of COVID-19 epidemic dynamics in Saudi Arabia using SIR-PSO and machine learning approaches | Saudi Arabia | Biomedical Databases | SARS-CoV-2 | Susceptible-Infected-Recovered-Particle-Swarm-Optimization Model, feed-forward artificial neural network models | Modelling disease incidence in population | N/A | N/A | N/A |
| Nayan (2022) | Coronavirus disease situation analysis and prediction using machine learning: a study on Bangladeshi population | Bangladesh | Longitudinal Survey Data | SARS-CoV-2 | MLP, SVR, Linear regression | Comparison of models/approaches; modelling disease incidence in population | N/A | N/A | N/A |
| Butaru (2022) | Resource Management through Artificial Intelligence in Screening Programs—Key for the Successful Elimination of Hepatitis C | Romania | Longitudinal Survey Data | Hepatitis | ANN | Modelling risk in population | Model Design | Just mentioned bias in general | Adressed incomplete data |
| Nazari (2022) | Evaluating Measles Incidence Rates Using Machine Learning and Time Series Methods in the Center of Iran, 1997-2020 | Iran | Longitudinal Survey Data | Measles | RF, LSSVM, LDA, NB, Logistic regression, ANN, SVM | Comparison of models/approaches; modselling risk in population | Model Implementation | Age, sex, ethnicity, location | variables adjusted for in regression |
| Alshabeeb (2022) | Machine Learning Techniques and Forecasting Methods for Analyzing and Predicting Covid-19 | Iraq | Biomedical Databases | SARS-CoV-2 | K-means | Modelling disease incidence in population AND comparison of models/approaches | N/A | N/A | N/A |
| Avirappattu (2022) | An optimized machine learning model for identifying socio-economic, demographic and health-related variables associated with low vaccination levels that vary across ZIP codes in California | USA | Biomedical Databases AND longitudinal survey data | SARS-CoV-2 | Linear regression, DT, SVM and GBR | Modelling risk in population and comparison of models/approaches | N/A | N/A | N/A |
| Absar (2022) | The efficacy of deep learning based LSTM model in forecasting the outbreak of contagious diseases | Bangladesh | Biomedical Databases | SARS-CoV-2 | LSTM | Modelling risk in population | Model Design | N/A | Any biasness of algorithm from the design point of view has been reduced by using LSTM networks |
| Abdullahi (2022) | Predicting diarrhoea outbreaks with climate change | South Africa | Biomedical Databases | Diarrheal Diseases | SVM, LSTM, and CNN | Modelling risk in population and comparison of models/approaches | N/A | N/A | N/A |
| An (2022) | Using the hybrid EMD-BPNN modelto predict the incidence of HIV in Dalian,Liaoning Province, China, 2004–2018 | China | Biomedical Databases | HIV | ANN | Modelling disease incidence in population AND comparison of models/approaches | Model Training | Socioeconomic Status/class (within populations) | N/A |
| Angeli (2022) | Modeling the effect of the vaccination campaign on the COVID-19 pandemic | United States | Biomedical Databases | SARS-CoV-2 | Neural Network | Evaluating effectiveness of intervention AND Modelling disease incidence in population | Model Training | Model does not take into consideration age, gender or geographic clustering | N/A |
| Abdullah (2022) | Predictions and visualization for confirmed, recovered and deaths COVID-19 cases in Iraq | Iraq | Biomedical Databases | SARS-CoV-2 | KNN and Linear regression | Comparison of models/approaches | N/A | N/A | N/A |
| Althomsons (2022) | Using Machine Learning Techniques and National Tuberculosis Surveillance Data to Predict Excess Growth in Genotyped Tuberculosis Clusters | United States | Biomedical Databases | Tuberculosis | Tree-based ensembles, SVM, regularized regression | Surveillance | N/A | N/A | N/A |
| Antweiler (2022) | Uncovering chains of infections through spatio-temporal and visualanalysis of COVID-19 contact traces | Germany | Biomedical Databases | SARS-CoV-2 | DT, RF, Naïve Bayes, SVM | Comparison of models/approaches AND Surveillance | Model Training | Data availability is dependent on the ability of the individuals being interviewed to recall contacts and symptoms as well as detailedness of the interview. | N/A |
| Babu (2022) | The mathematical and machine learning models to forecast the COVID-19 outbreaks in Bangladesh | Bangladesh | Longitudinal Survey Data | SARS-CoV-2 | SEIRD model, SEIRD based ML model, FBProphet ML forecasting model | Modelling risk in population; Comparison of models | N/A | N/A | N/A |
| Li (2022) | Segregation Predicts COVID-19 Fatalities in Less Densely Populated Counties | USA | Longitudinal Survey Data | SARS-CoV-2 | DT | Modelling disease incidence in population | Model Implementation | Sex, ethnicity, SES | incorporated into the ML and checked for associations between variables |
| Mohan (2021) | AN approach to forecast impact of COVID-19 using supervised machine learning model | India, global | Biomedical Databases | SARS-CoV-2 | Autoregressive, Moving average model, EAMA model | Comparison of models/approaches | N/A | N/A | N/A |
| Mohammadi (2021) | Comparative study of linear regression and SIR models of COVID-19 propagation in Ukraine before vaccination | Ukraine | Biomedical Databases | SARS-CoV-2 | SIR | Comparison of models/approaches | N/A | N/A | N/A |
| Mohimant (2021) | Convolutional neural networks and temporal CNNs for COVID-19 forecasting in France | France | Biomedical Databases | SARS-CoV-2 | Temporal Convolutional Network, CNN | Comparison of models/approaches | Model Implementation | N/A | N/A |
| Migriño (2021) | Using machine learning to create a decision tree model to predict outcomes of COVID-19 cases in the Philippines | Philippines | Biomedical Databases | SARS-CoV-2 | DT | Modelling disease incidence in population | Model Training | N/A | N/A |
| Naeem (2021) | Comparative analysis of machine learning approaches to analyze and predict the COVID-19 outbreak | Worldwide | Longitudinal Survey Data | SARS-CoV-2 | SVM, RF, K-Nearest Neighbor and ANN | Comparison of models/approaches; modelling disease incidence in population | N/A | N/A | N/A |
| Omran (2021) | Applying Deep Learning Methods on Time-Series Data for Forecasting COVID-19 in Egypt, Kuwait, and Saudi Arabia | Egypt, Saudi Arabia, and Kuwait | Longitudinal Survey Data | SARS-CoV-2 | LSTM, gated recurrent unit (GRU) | Comparison of models/approaches | N/A | N/A | N/A |
| Alkhammash (2021) | Novel PredictionModel for COVID-19 in Saudi Arabia Based on an LSTM Algorithm | Saudi Arabia | Biomedical Databases | SARS-CoV-2 | DNN, LSTM, transformer (all neural networks) | Modelling disease incidence in population AND comparison of models/approaches | N/A | N/A | N/A |
| Ayoobi (2021) | Time series forecasting of new cases and new deaths rate for COVID-19 using deep learning methods | Australia, Iran | Longitudinal Survey Data | SARS-CoV-2 | LSTM, Conv-LSTM, GRU, BI-LSTM, Bi-Conv-LSTM and Bi-GRU | Comparison of models/approaches | Model Training | Preduction bias | rely on the data collected from the population in a dynamic manner and use them during the training and prediction of our neural network-based model. Therefore, our model is able to adapt to the changing dynamics of the population on the fly, which reduces the bias in its prediction |
| Prasanth (2021) | Forecasting spread of COVID-19 using google trends: A hybrid GWO-deep learning approach | India | Social Media | SARS-CoV-2 | LSTM | Modelling disease incidence in population AND comparison of models/approaches | N/A | N/A | N/A |
| Milivojević (2021) | Long Short-Term Memory Prediction for COVID19 Time Series | Republic of Serbia | Biomedical Databases | SARS-CoV-2 | LSTM, ARIMA | Modelling disease incidence in population | N/A | N/A | N/A |
| Ossa (2021) | A Hybrid Model for COVID-19 Monitoring and Prediction | Colombia | Longitudinal Survey Data | SARS-CoV-2 | SIR, LSTM | Modelling disease incidence in population | Model Implementation | Age, gender | No |
| McCoy (2021) | Ensemble machine learning of factors influencing COVID‑19 across US counties | USA | Biomedical Databases | SARS-CoV-2 | Linear regression | Modelling risk in population | Model Implementation | N/A | N/A |
| Çaparoglu (2021) | To restrict or not to restrict? Use of artificial neural network to evaluate the effectiveness of mitigation policies: A case study of Turkey | Turkey | Biomedical Databases | SARS-CoV-2 | ANN | Modelling disease incidence in population | N/A | N/A | N/A |
| Al-Ridha (2021) | Expecting confirmed and death cases of covid-19 in Iraq by utilizing backpropagation neural network | Iraq | Biomedical Databases | SARS-CoV-2 | ANN | Modelling disease incidence in population | N/A | N/A | N/A |
| Ly (2021) | A COVID-19 forecasting system using adaptive neuro-fuzzy inference | UK | Longitudinal Survey Data | SARS-CoV-2 | Adaptive Neuro-Fuzzy Inference System (ANFIS) | Modelling disease incidence in population | N/A | N/A | N/A |
| Khayyat (2021) | Time Series Facebook Prophet Model and Python for COVID-19 Outbreak Prediction | Saudi Arabia | Biomedical Databases | SARS-CoV-2 | Prophet (additive regression model) | Modelling disease incidence in population | N/A | N/A | N/A |
| Khan (2021) | COVID-19 in China: Risk Factors and R0 Revisited | China | Biomedical Databases | SARS-CoV-2 | Stochastic transmission model | Surveillance AND modelling disease incidence in population | Model Training | Reporting bias | N/A |
| Khakharia (2021) | Outbreak Prediction of COVID‑19 for Dense and Populated Countries Using Machine Learning | India | Biomedical Databases | SARS-CoV-2 | ARMA, ARIMA, SVR, Linear Regressor polynomial (LRP), Bayesian Ridge Regression (BRR), Linear regression, Random Forest Regressor (RFR), Holt-Winter Exponential Smoothing (HW), and Extreme Gradient Boost Regressor (XGB) | Modelling disease incidence in population AND comparison of models/approaches | N/A | N/A | N/A |
| Oshinubi (2021b) | Approach to COVID-19 time series data using deep learning and spectral analysis methods | France, Russia, Turkey, India, USA, Brazil and UK | Longitudinal Survey Data | SARS-CoV-2 | Extreme machine learning (ELM), multilayer perceptron (MLP), LSTM, gated recurrent unit (GRU), CNN and DNN | Modelling disease incidence in population | N/A | N/A | N/A |
| Oshinubi (2021a) | Data Analysis and Forecasting of COVID-19 Pandemic in Kuwait Based on Daily Observation and Basic Reproduction Number Dynamics | Kuwait | Longitudinal Survey Data | SARS-CoV-2 | ARIMA model, Exponential smoothing model, Holt’s method, Prophet forecasting model, log-linear, polynomial and support vector regressions | Comparison of models/approaches; modelling disease incidence in population | N/A | N/A | N/A |
| Kao (2021) | Early prediction of coronavirus disease epidemic severity in the contiguous United States based on deep learning | USA | Biomedical Databases | SARS-CoV-2 | Convolutional Autoencoder and AL-CNN (convolutional autoencoder with LSTM) | Modelling disease incidence in population AND comparison of models/approaches | N/A | N/A | N/A |
| Kaliappan (2021) | Performance Evaluation of Regression Models for the Prediction of the COVID-19 Reproduction Rate | India | Biomedical Databases | SARS-CoV-2 | Random Forest, Gradient Boosting, XGBOOST, KNN, SVR | Modelling disease incidence in population AND comparison of models/approaches | N/A | N/A | N/A |
| Price (2021) | Predicting increases in COVID-19 incidence to identify locations for targeted testing in West Virginia: A machine learning enhanced approach | USA | Biomedical Databases AND epidemiological statistics | SARS-CoV-2 | LSTM | Modelling disease incidence in population AND comparison of models/approaches | Model Training | Bias towards rural counties with a low incidence | Decreasing number of tests |
| Lounis (2021) | Predictive models for COVID-19 cases, deaths and recoveries in Algeria | Algeria | Longitudinal Survey Data | SARS-CoV-2 | Gompertz model, logistic model, Bertalanffy model and inverse artificial neural network (ANNi). | Comparison of models/approaches; modelling disease incidence in population | N/A | N/A | N/A |
| Li (2021) | Unraveling the dynamic importance of county‑level features in trajectory of COVID‑19 | USA | Longitudinal Survey Data | SARS-CoV-2 | RF | Modelling disease incidence in population | Model Implementation | SES | No |
| Al-Qaness (2021) | Efficient artificial intelligence forecasting models for COVID-19 outbreak in Russia and Brazil | Russia and Brazil | Biomedical Databases | SARS-CoV-2 | ANFIS | Modelling disease incidence in population AND comparison of models/approaches | N/A | N/A | N/A |
| Zheng (2021) | Early Warning Method for Public Health Emergency Under Artificial Neural Network in the Context of Deep Learning | China | Social Media, Biomedical Databases | Tuberculosis | ANN, CNN, Hybrid ANN and CNN model | Surveillance | N/A | N/A | N/A |
| Zhan (2021) | Random-Forest-Bagging Broad Learning System With Applications for COVID-19 Pandemic | Global, 184 countries | Biomedical Databases | SARS-CoV-2 | Linear regression, KNN, DT, adaptive boosting (Ada), RF, gradient boosting DT (GBDT), SVR, extra trees (ETs) regressor, CatBoost (CAT), LightGBM (LGB), XGBoost (XGB), and BLS | Comparison of models/approaches | N/A | N/A | N/A |
| Zawbaa (2021) | A study of the possible factors affecting COVID-19 spread, severity and mortality and the effect of social distancing on these factors: Machine learning forecasting model | Global | Biomedical Databases | SARS-CoV-2 | MLP | Modelling disease incidence in population | N/A | N/A | N/A |
| Zaidi (2021) | Future Prediction of COVID-19 Vaccine Trends Using a Voting Classifier | Global | Social Media | SARS-CoV-2 | RF, SVM, DT, KNN, and ANN | Evaluating attitude towards an intervention | N/A | N/A | N/A |
| Yu (2021) | A COVID-19 Pandemic Artificial Intelligence–Based System With Deep Learning Forecasting and Automatic Statistical Data Acquisition: Development and Implementation Study | Global, 171 countries | Biomedical Databases, Policy trackers | SARS-CoV-2 | LSTM, FNN, MLP, neural network | Modelling disease incidence in population | N/A | N/A | N/A |
| Yeung (2021) | Machine Learning–Based Prediction of Growth in Confirmed COVID-19 Infection Cases in 114 Countries Using Metrics of Nonpharmaceutical Interventions and Cultural Dimensions: Model Development and Validation | Global, 114 countries | Biomedical Databases, Policy trackers | SARS-CoV-2 | Ridge regression, DT regression, SVR, RF regression, AdaBoost Regression | Modelling disease incidence in population | N/A | N/A | N/A |
| Xylogiannopoulos (2021) | COVID-19 pandemic spread against countries’ non-pharmaceutical interventions responses: a data-mining driven comparative study | Global, 48 countries | Biomedical Databases | SARS-CoV-2 | General Purpose Sequence Clustering Algorithm | Evaluating effectiveness of intervention | N/A | N/A | N/A |
| Watson (2021) | Pandemic velocity: Forecasting COVID-19 in the US with a machine learning & Bayesian time series compartmental model | USA | Biomedical Databases | SARS-CoV-2 | RF | Modelling disease incidence in population | N/A | N/A | N/A |
| Vadyala (2021) | Prediction of the number of COVID-19 confirmed cases based on K-means-LSTM | USA | Biomedical Databases | SARS-CoV-2 | SEIR, K-Means-LSTM | Modelling disease incidence in population | N/A | N/A | N/A |
| Almazroi (2021) | COVID-19 Cases Prediction in Saudi Arabia Using Tree-based Ensemble Models | Saudi Arabia | Biomedical Databases | SARS-CoV-2 | Gradient Tree Boosting (GB), RF, Extreme Gra- dient Boosting (XGBoost) and Voting Regressor (VR). | Modelling disease incidence in population AND comparison of models/approaches | Model Design | N/A | N/A |
| Toharudin (2021) | Indonesia in Facing New Normal: An Evidence Hybrid Forecasting of COVID-19 Cases Using MLP, NNAR and ELM | Indonesia | Biomedical Databases | SARS-CoV-2 | NNAR, ELM, MLP | Modelling disease incidence in population | N/A | N/A | N/A |
| Toh (2021) | Improving national level spatial mapping of malaria through alternative spatial and spatio-temporal models | Burkino Faso, Mali, Malawi, Nigeria, Uganda | Biomedical Databases | Malaria | Stepwise logistic regression, Generalized additive model (GAM), Gaussian process model usuing SPDE-INLA, Gradient boosted trees | Modelling disease incidence in population | N/A | N/A | N/A |
| Tiwari (2021) | Using machine learning to develop a novel COVID-19 Vulnerability Index (C19VI) | USA | Biomedical Databases | SARS-CoV-2 | RF | Modelling risk in population | N/A | N/A | N/A |
| Snider (2021) | Identification of Variable Importance for Predictions of Mortality From COVID-19 Using AI Models for Ontario, Canada | Canada | Biomedical Databases | SARS-CoV-2 | XGBoost, ANN, and RF | Modelling risk in population | N/A | N/A | N/A |
| Mahmud (2021) | Predicting Spread, Recovery and Death Due to COVID-19 using a Time-Series Model (Prophet) | Brazil, USA, Canada, UK, Spain, Italy, Singapore, Japan, China and South Korea | Longitudinal Survey Data | SARS-CoV-2 | Prophet Forecasting Model, SVM, LSTM and ARIMA | Comparison of models/approaches; modelling disease incidence in population | N/A | N/A | N/A |
| Ahmad (2021) | An Improved COVID-19 Forecasting by Infectious Disease Modelling Using Machine Learning | Saudi Arabia, Kuwait, Bahrain, and the UAE | Biomedical Databases | SARS-CoV-2 | Linear regression, PR, SVR, SIR, LSTM, and Bi-LSTM | Comparison of models/approaches | N/A | N/A | N/A |
| Shetty (2021) | Forecasting of COVID 19 Cases in Karnataka State using Artificial Neural Network (ANN) | India | Biomedical Databases | SARS-CoV-2 | ANN | Comparison of models/approaches | Model Design | N/A | this model has the ability to adjusts synaptic weights and biases to best fit the given environment. |
| Shastri (2021b) | Deep-LSTM ensemble framework to forecast Covid-19: an insight to the global pandemic | India | Biomedical Databases | SARS-CoV-2 | LSTM | Comparison of models/approaches | N/A | N/A | N/A |
| Shastri (2021a) | CoBiD-net: A tailored deep learning ensemble model for time series forecasting of covid-19 | India and Brazil | Biomedical Databases | SARS-CoV-2 | Bi-directional LSTM, Convolutional LSTM , CoBiD ensemble (a type of RNN) | Comparison of models/approaches | N/A | N/A | N/A |
| Satu (2021) | Short-Term Prediction of COVID-19 Cases Using Machine Learning Models | Bangladesh | Biomedical Databases | SARS-CoV-2 | Linear regression, Polynomial Regression (PR),  SVR, MLP, Polynomial Multi-Layer perceptron (Poly-MLP), and Prophet algorithm | Modelling disease incidence in population AND comparison of models/approaches | N/A | N/A | N/A |
| Said (2021) | Predicting COVID-19 cases using bidirectional LSTM on multivariate time series | Qatar | Biomedical Databases and SDH data | SARS-CoV-2 | Bidirectional Long Short-Term Memory | Modelling disease incidence in population | N/A | N/A | N/A |
| Sahai (2021) | A machine learning model for nowcasting epidemic incidence | USA | Biomedical Databases | SARS-CoV-2 | RF | Modelling disease incidence in population | N/A | N/A | N/A |
| Majhi (2021) | Analysis and prediction of COVID-19 trajectory: A machine learning approach | India | Longitudinal Survey Data | SARS-CoV-2 | Nonlinear Regression, Decision Tree based regression, and RF | Comparison of models/approaches; modelling disease incidence in population | N/A | N/A | N/A |
| Kalantari (2021) | Forecasting COVID-19 pandemic using optimal singular spectrum analysis | Iran | Biomedical Databases | SARS-CoV-2 | ARIMA, Fractional ARIMA (ARFIMA), Exponential Smoothing, TBATS, NNAR | Modelling disease incidence in population AND comparison of models/approaches | N/A | N/A | N/A |
| Abreu (2021) | A Two-Step Polynomial and Nonlinear Growth Approach for Modeling COVID-19 Cases in Mexico | Mexico | Longitudinal Survey Data | SARS-CoV-2 | Polynomial model, Nonlinear growth models, including logistic and Gompertz | Modelling disease incidence in population; comparison of models | N/A | N/A | N/A |
| Ayoub (2021) | Classification and Categorization of COVID-19 Outbreak in Pakistan | Pakistan | Longitudinal Survey Data | SARS-CoV-2 | DT, NB, LR, and SMV | Modelling disease incidence in population | N/A | N/A | N/A |
| Berhich (2021) | Multiple Output and Multi-Steps Prediction of COVID-19 Spread Using Weather and Vaccination Data | Morocco, Algeria, Saudi Arabia, USA, China, and Iceland | Longitudinal Survey Data | SARS-CoV-2 | RF, MLP, LSTM, CNN | Comparison of models/approaches; modelling disease incidence in population | N/A | N/A | N/A |
| Hamadneh (2021a) | Artificial Neural Networks for Prediction of COVID-19 in Saudi Arabia | Saudia Arabia | Biomedical Databases | SARS-CoV-2 | ANN | Modelling disease incidence in population | N/A | N/A | N/A |
| Harvey (2021) | Predicting malaria epidemics in Burkina Faso with machine learning | Burkina Faso | Biomedical Databases | Malaria | RF | Modelling disease incidence in population | Model Implementation | N/A | N/A |
| Arik (2021) | A prospective evaluation of AI-augmented epidemiology to forecast COVID-19 in the USA and Japan | USA and Japan | Biomedical Databases | SARS-CoV-2 | XGBoost | Evaluating effectiveness of intervention AND Modelling disease incidence in population | Model Implementation | Sex, income, age, population density, and ethnicity | Age, sex, income, population density, and ethnicity are investigated for both the USA and Japanese models. These variables are chosen based on known biases in how COVID-19 has affected different demographics, as well as how they may affect healthcare access. To investigate these relationships, differences changes in the MAPE of the forecasts were compared to the demographics from each geographical region (counties for the USA and prefectures for Japan). The authors also applied regularization to improve generalization to unseen future data. |
| Arlis (2021) | Machine Learning Algorithms for Predictingthe Spread of Covid‒19 in Indonesia | Indonesia | Biomedical Databases | SARS-CoV-2 | K-means, KNN, DT (ID3) | Evaluating effectiveness of intervention AND Modelling disease incidence in population | N/A | N/A | N/A |
| Dairi (2021) | Comparative study of Machine learning methods for COVID-19 transmission forecasting | Brazil, France, India, Mexico, USA | Biomedical Databases | SARS-CoV-2 | Hybrid LSTM-CNN, LSTM, CNN, RBM, GAN-DNN, GAN-GRU, and LSTM-CNN | Modelling disease incidence in population | N/A | N/A | N/A |
| Hamadneh (2021b) | Using Artificial Neural Network with Prey Predator Algorithm for Prediction of the COVID-19: The Case of Brazil and Mexico | Brazil and Mexico | Biomedical Databases | SARS-CoV-2 | ANN with a prey predator algorithm (PPA) | Modelling disease incidence in population | Model Implementation | N/A | N/A |
| Likassa (2021) | Predictive models on COVID 19: What Africans should do? | Africa | Longitudinal Survey Data | SARS-CoV-2 | Logarithmic, compound, growth, exponential, logistic, quadratic, cubic | Comparison of models/approaches; modelling disease incidence in population | N/A | N/A | N/A |
| Gupta (2021c) | Prediction of COVID-19 Confirmed, Death, and Cured Cases in India Using Random Forest Model | India | Biomedical Databases | SARS-CoV-2 | RF, linear model, SVM, DT, neural network | Modelling disease incidence in population | N/A | N/A | N/A |
| Gupta (2021b) | Comparative analysis of epidemiological models for COVID-19 pandemic predictions | Global | Biomedical Databases | SARS-CoV-2 | Gompertz, Logistic, and Exponential growth curve fitting models. Two mathematical models (SEIR and IDEA), two forcasting models (Holt's exponential and ARIMA) and four machine/deep learning models (Neural Network, LTSM Networks, GANs, and Random Forest) | Modelling disease incidence in population | N/A | N/A | N/A |
| Devaraj (2021) | Forecasting of COVID-19 cases using deep learning models: Is it reliable and practically significant? | Global | Biomedical Databases | SARS-CoV-2 | ARIMA, LSTM, SLSTM, and Prophet model | Modelling disease incidence in population | N/A | N/A | N/A |
| Gupta (2021a) | AI-Enabled COVID-19 Outbreak Analysis and Prediction: Indian States vs. Union Territories | India | Biomedical Databases | SARS-CoV-2 | Polynomial regression, DT, RF | Modelling disease incidence in population | N/A | N/A | N/A |
| Arora (2021) | Prediction and forecasting of COVID-19 outbreak using regression and ARIMA models | India | Biomedical Databases | SARS-CoV-2 | ARIMA, and regression models | Modelling disease incidence in population AND comparison of models/approaches | N/A | N/A | N/A |
| ArunKumar (2021) | Forecasting the dynamics of cumulative COVID-19 cases (confirmed,recovered and deaths) for top-16 countries using statistical machinelearning models: Auto-Regressive Integrated Moving Average (ARIMA)and Seasonal Auto-Regressive Integrated Moving Average (SARIMA) | USA | Biomedical Databases | SARS-CoV-2 | ARIMA and SARIMA | Modelling disease incidence in population AND comparison of models/approaches | N/A | N/A | N/A |
| Gray (2021) | e- sults revealed that coughing had the highest positive correlation with the positive results of COVID- 19 test followed by the duration of having COVID-19 signs and symptoms, exposure to infected individuals, age, muscle pain, recent infection by COVID-19 virus, fever, respiratory distress, loss of smell or taste, nausea, anorexia, headache, vertigo, CT symptoms in lung scans, diabetes and hyper- tension. | USA | Biomedical Databases | SARS-CoV-2 | Various regression models | Modelling disease incidence in population | N/A | N/A | N/A |
| Nikparvar (2021) | Spatio‑temporal prediction of the COVID‑19 pandemic in US counties: modeling with a deep LSTM neural network | USA | Longitudinal Survey Data | SARS-CoV-2 | MTS-LSTM | Modelling disease incidence in population | N/A | N/A | N/A |
| Gerts (2021) | “Thought I’d Share First” and Other Conspiracy Theory Tweets from the COVID-19 Infodemic: Exploratory Study | Global | Social Media | SARS-CoV-2 | RF | Surveillance | N/A | N/A | N/A |
| Elkhadrawi (2021) | Machine Learning Classification of False-Positive Human Immunodeficiency Virus Screening Results | USA | Biomedical Databases | HIV | SVM algorithm | Surveillance | N/A | N/A | N/A |
| Kafieh (2021) | COVID-19 in Iran: Forecasting Pandemic Using Deep Learning | Iran | Biomedical Databases | SARS-CoV-2 | RF, multilayer perceptron, LSTM | Modelling disease incidence in population AND comparison of models/approaches | N/A | N/A | N/A |
| Elsheikh (2021) | Deep learning-based forecasting model for COVID-19 outbreak in Saudia Arabia | Saudia Arabia | Biomedical Databases | SARS-CoV-2 | LSTM | Modelling disease incidence in population | N/A | N/A | N/A |
| Fan (2021) | Estimating global burden of COVID-19 with disability-adjusted life years and value of statistical life metrics | Global | Biomedical Databases | SARS-CoV-2 | K-means | Surveillance | N/A | N/A | N/A |
| Marin-Gomez (2021) | Assessing the likelihood of contracting COVID-19 disease based on a predictive tree model: A retrospective cohort study | Spain | EMRs | SARS-CoV-2 | Logistic model, decision tree | Modelling disease incidence in population; comparison of models | Model Implementation | age, sex, SES | Variables accounted for in models. |
| Hariharan (2021) | Random forest regression analysis on combined role of meteorological indicators in disease dissemination in an Indian city: A case study of New Delhi | India | Biomedical Databases | SARS-CoV-2 | RF | Modeling risk in population | Model Implementation | N/A | N/A |
| Fernandes (2021) | Predicting COVID-19 Vaccination Intention: The Determinants of Vaccine Hesitancy | Portugal | Biomedical Databases | SARS-CoV-2 | ANN | Vaccine Intention | N/A | N/A | N/A |
| Cuomo (2021) | A longitudinal and geospatial analysis of COVID-19 tweets during the early outbreak period in the United States | USA | Social Media | SARS-CoV-2 | SVM Classifier | Modelling disease incidence in population | N/A | N/A | N/A |
| Castillo-Olea (2021) | Early Stage Identification of COVID-19 Patients in Mexico Using Machine Learning: A Case Study for the Tijuana General Hospital | Mexico | EMRs | SARS-CoV-2 | Logistic regression, neural networks | Modelling risk in population | N/A | N/A | N/A |
| Chen (2021) | Model-based forecasting for Canadian COVID-19 Data | Canada | Biomedical Databases | SARS-CoV-2 | Smooth transition auto-regressive (STAR), neural network, and susceptible-infected-removed (SIR) models | Modelling disease incidence in population | N/A | N/A | N/A |
| Andelic (2021) | Estimation of COVID-19 epidemic curves using genetic programming algorithm | China, Italy, Spain, and USA | Biomedical Databases | SARS-CoV-2 | Genetic Programming Algorithm | Evaluating effectiveness of intervention AND Modelling disease incidence in population | N/A | N/A | N/A |
| Kuo (2021) | Evaluating the impact of mobility on COVID-19 pandemic with machine learning hybrid predictions | USA | Longitudinal Survey Data | SARS-CoV-2 | EN model; principal components regression (PCR); partial least squares regression, KNN; regression tree; RF; gradient boosted tree; ANN | Modelling disease incidence in population; comparison of models | N/A | N/A | N/A |
| Bloise (2021) | Predicting the spread of COVID-19 in Italy using machine learning: Do socio-economic factors matter? | Italy | Longitudinal Survey Data | SARS-CoV-2 | OLS, LASSO, RIDGE regression | Modelling disease incidence in population | N/A | N/A | N/A |
| Aravind (2021) | Predicting COVID-19 Cases in Indian States using Random Forest Regression | India | Biomedical Databases | SARS-CoV-2 | RF | Modelling disease incidence in population | Model Implementation | N/A | N/A |
| Jung (2021) | Predicting the effective reproduction number of COVID-19: inference using human mobility, temperature, and risk awareness | Japan | Biomedical Databases | SARS-CoV-2 | Linear regression | Modelling disease incidence in population | N/A | N/A | N/A |
| Jamshidnezhad (2021) | The role of ambient parameters on transmission rates of the COVID-19 outbreak: A machine learning model | Iran | Biomedical Databases AND climatic factors | SARS-CoV-2 | ANN | Modelling risk in population | N/A | N/A | N/A |
| Huamaní (2021) | Analysis and Prediction of Recorded COVID-19 Infections in the Constitutional Departments of Peru using Specialized Machine Learning Techniques | Peru | Biomedical Databases | SARS-CoV-2 | Not specified | Modelling disease incidence in population | N/A | N/A | N/A |
| Hashim (2021) | INTEGRATING DATA WAREHOUSE AND MACHINE LEARNING TO PREDICT ON COVID-19 PANDEMIC EMPIRICAL DATA | USA, India, Brazil, Russia, Spain, Argentina, France, Colombia, Peru, and Mexico | Biomedical Databases | SARS-CoV-2 | LSTM, DT, and ARIMA | Modelling disease incidence in population | Model Implementation | N/A | N/A |
| Cui (2021) | A two-layer nested heterogeneous ensemble learning predictive method for COVID-19 mortality | Global, 79 countries | Biomedical Databases | SARS-CoV-2 | Heterogenous ensemble learning-based prediction method, Linear regression, SVRn, Classification and regression tree, Extreme learning machine, RF, XGBoost, LightGBM, and LSTM. | Predicting COVID-19 Mortality | N/A | N/A | N/A |
| Zeroual (2020) | Deep learning methods for forecasting COVID-19 time-Series data: A comparative study | Italy, Spain, France, China, USA, and Australia | Biomedical Databases | SARS-CoV-2 | RNN, LSTM, Bidirectional LSTM (BiLSTM), Gated recurrent units (GRUs) and Variational AutoEncoder (VAE) algorithims | Comparison of models/approaches | N/A | N/A | N/A |
| Tamang (2020) | Forecasting of Covid-19 cases based on prediction using artificial neural network curve fitting technique | India, USA, France, UK, China, South Korea | Biomedical Databases | SARS-CoV-2 | ANN | Modelling disease incidence in population | N/A | N/A | N/A |
| Fang (2020) | Forecasting incidence of infectious diarrhea using random forest in Jangsu Province, China | China | Biomedical Databases | Diarrheal Diseases | RF | Modelling disease incidence in population | N/A | N/A | N/A |
| Sun (2020) | Forecasting the long-term trend of COVID-19 epidemic using a dynamic model | China | Biomedical Databases | SARS-CoV-2 | Dynamic-Susceptible-Exposed-Infective-Quarantined (D-SEIQ) a modified Susceptible-Exposed-Infective-Recovered (SEIR) model | Modelling disease incidence in population | N/A | N/A | N/A |
| Zhu (2020) | Learning from Large-Scale Wearable Device Data for Predicting the Epidemic Trend of COVID-19 | China, South-Central Europe | User wearable data, Biomedical Databases | SARS-CoV-2 | Heterogenous neural network regression model combining sparse categorical features and dense numerical featured (CDNet) | Surveillance | N/A | N/A | N/A |
| Torrealba-Rodriguez (2020) | Modelling and prediction of COVVID-19 in Mexico applying mathematical and computational models | Mexico | Biomedical Databases | SARS-CoV-2 | ANN | Modelling disease incidence in population | N/A | N/A | N/A |
| Tuli (2020) | Predicting the growth and trend of COVID-19 pandemic using machine learning and cloud computing | Global | Biomedical Databases | SARS-CoV-2 | Regression | Modelling disease incidence in population | N/A | N/A | N/A |
| Fakhry (2020) | Tracking Coronavirus Pandemic Diseases using Social Media: A Machine Learning Approach | USA | Social Media | SARS-CoV-2 | SVM, Naïve Bayes Algorithm | Modelling risk in population | N/A | N/A | N/A |
| Wang (2020) | Prediction of epidemic trends in COVID-19 with logistic model and machine learning technics | Global | Biomedical Databases | SARS-CoV-2 | Logistic model, FbProhpet model | Modelling disease incidence in population | N/A | N/A | N/A |
| Utsunomiya (2020) | Growth Rate and Acceleration Analysis of the COVID-19 Pandemic Reveals the Effect of Public Health Measures in Real Time | Worldwide | Biomedical Databases | SARS-CoV-2 | Moving Regression Model, Hidden Markov Model (HMM) | Modelling disease incidence in population | N/A | N/A | N/A |
| Liu (2020) | Real-Time Forecasting of the COVID-19 Outbreak in Chinese Provinces: Machine Learning Approach Using Novel Digital Data and Estimates From Mechanistic Models | China | Longitudinal Survey Data | SARS-CoV-2 | Global epidemic and mobility model (GLEAM), ARGONet | Modelling disease incidence in population | N/A | N/A | N/A |
| Yesilkanat (2020) | Spatio-temporal estimation of the daily cases of COVID-19 in worldwide using random forest machine learning algorithm | Global, 190 countries | Biomedical Databases | SARS-CoV-2 | RF | Modelling disease incidence in population | N/A | N/A | N/A |
| Li (2020) | A comparative study on the prediction of the BP artificial neural network model and the ARIMA model in the incidence of AIDS | China | Longitudinal Survey Data | HIV | ARIMA; BP-ANN | Comparison of models/approaches | N/A | N/A | N/A |
| Warsito (2020) | Short Term Prediction of COVID-19 Cases By Using Various Types of Neural Network Model | Indonesia | Biomedical Databases | SARS-CoV-2 | (1) Feed Forward Neural Network (FFNN), (2) Cascade Forward Neural Network (CFNN), (3) General Regression Neural Network, and (4) Recurrent Neural Network (RNN) | Modelling disease incidence in population | N/A | N/A | N/A |
| Ksantini (2020) | Artificial Intelligence Prediction Algorithms for Future Evolution of COVID-19 Cases | France, Italy, Spain, USA, Tunisia, Algeria, Morocco and Belgium. | Longitudinal Survey Data | SARS-CoV-2 | RF, Xgboost, BiLSTM | Modelling disease incidence in population | N/A | N/A | N/A |
| Brown (2020) | Data‑driven malaria prevalence prediction in large densely populated urban holoendemic sub‑Saharan West Africa | Nigeria | Longitudinal Survey Data | Malaria | GLM, EM and SVM | Modelling disease incidence in population | N/A | N/A | N/A |
| Weiczorek (2020) | Real-time neural network based predictor for cov19 virus spread | Global | Biomedical Databases | SARS-CoV-2 | ANN | Modelling disease incidence in population | N/A | N/A | N/A |
| Weiczorek (2020) | Neural network powered COVID-19 spread forecasting model | Global | Biomedical Databases | SARS-CoV-2 | Classic ANN | Modelling disease incidence in population | N/A | N/A | N/A |
| Yudistira (2020) | COVID-19 Growth Prediction using Multivariate Long Short Term Memory | Global | Biomedical Databases | SARS-CoV-2 | LTSM, vector autoregression (VAR), RNN | Modelling disease incidence in population | N/A | N/A | N/A |
| Guo (2020) | Prediction of hepatitis E using machine learning models | China | Biomedical Databases | Hepatitis | ARIMA, SVM, and LSTM | Modelling disease incidence in population | N/A | N/A | N/A |
| Li (2020) | Study on Prediction Model of HIV Incidence Based on GRU Neural Network Optimized by MHPSO | China | Longitudinal Survey Data | HIV | BPNN model, RNN model, LSTM model and MHPSO-GRU model | Comparison of models/approaches | N/A | N/A | N/A |
| Cuomo (2020) | Characterising communities impacted by the 2015 Indiana HIV outbreak: A big data analysis of social media messages associated with HIV and substance abuse | USA | Social Media | HIV | Linear regression | Modelling risk in population | N/A | N/A | N/A |
| Kafieh (2020) | Isfahan and Covid-19: Deep spatiotemporal representation | Iran | Biomedical Databases and SDH data | SARS-CoV-2 | RF, multilayer perceptron, CNN, XGBoost | Modelling disease incidence in population AND comparison of models/approaches | N/A | N/A | N/A |
| Cai (2020) | Identification and characterization of tweets related to the 2015 Indiana HIV outbreak: A retrospective infoveillance study | USA | Social Media | HIV | NLP and Biterm Topic Mode (BTM) | Surveillance | N/A | N/A | N/A |
| Liu (2020) | Predicting and analyzing the COVID-19 epidemic in China: Based on SEIRD, LSTM and GWR models | China | Longitudinal Survey Data | SARS-CoV-2 | SEIRD, LSTM, GWR (Geographically Weighted Regression) model | Comparison of models/approaches; modelling disease incidence in population | N/A | N/A | N/A |
| Gupta (2020a) | Prediction of COVID-19 trends in Europe using generalized regression nerual network optimized by flower pollination algorithm | European Continent | Biomedical Databases | SARS-CoV-2 | Flower polination algorithm combined with generalized regression neural network (FPA-GRNN), SVM, and non-linear regression | Modelling disease incidence in population | N/A | N/A | N/A |
| Jung (2020) | Real-World Implications of a Rapidly Responsive COVID-19 Spread Model with Time-Dependent Parameters via Deep Learning: Model Development and Validation | Korea | Biomedical Databases | SARS-CoV-2 | Neural network | Comparison of models/approaches | N/A | N/A | N/A |
| Amar (2020) | Prediction of the final size for COVID-19 epidemic using machine learning: A case study of Egypt | Egypt | Biomedical Databases | SARS-CoV-2 | Logistic regression | Modelling disease incidence in population | N/A | N/A | N/A |
| Alzahrani (2020) | Forecasting the spread of the COVID-19 pandemic in Saudi Arabia using ARIMA prediction model under current public health interventions | Saudi Arabia | Biomedical Databases | SARS-CoV-2  -19 | Autoregressive Model and Moving Average | Modelling disease incidence in population | N/A | N/A | N/A |
| Pereira (2020) | Forecasting Covid-19 Dynamics in Brazil: A Data Driven Approach | Brazil | Longitudinal Survey Data | SARS-CoV-2 | SIR, SEIR, and SIRASD, LSTM, LSTM-SAE | Modelling disease incidence in population | N/A | N/A | N/A |
| Huang (2020) | Novel spatiotemporal feature extraction parallel deep neural network for forecasting confirmed cases of coronavirus disease 2019 | Taiwan | Biomedical Databases | SARS-CoV-2 | COVID-19Net (combines 1D convolutional neural network, 2D convolutional neural network, and bidirectional gated recurrent units); CNN, GRU, and CNN-GRU | Modelling disease incidence in population | N/A | N/A | N/A |
| Marvel (2020) | The COVID-19 Pandemic Vulnerability Index (1 PVI) Dashboard: Monitoring county-level vulnerability using visualization, statistical modeling, and machine learning | USA | Biomedical Databases | SARS-CoV-2 | Linear regression | Evaluating effectiveness of intervention | Model Design | A constant underreporting bias across counties would be absorbed into the intercept and would otherwise produce valid coefficient estimates for the predictors. Analysis (iv) may provide important clues about the death risk as including cases in the denominator removes a large portion of the stochastic variation. | Moreover, for all analyses, we used the proportion of the state population that has been tested as a predictor to account for additional sources of bias. |
| Mehta (2020) | Early Stage Machine Learning–Based Prediction of US County Vulnerability to the COVID-19 Pandemic: Machine Learning Approach | USA | Biomedical Databases | SARS-CoV-2 | XGBoost | Modelling disease incidence in population | Model Implementation | N/A | N/A |
| Melin (2020) | Multiple Ensemble Neural Network Models with Fuzzy Response Aggregation for Predicting COVID-19 Time Series: The Case of Mexico | Mexico | Biomedical Databases | SARS-CoV-2 | ANN | Comparison of models/approaches | N/A | N/A | N/A |
| Alsuwaiket (2020) | Predicting the Covid-19 spread, recoveries and mortalities rates in Saudi Arabia using ANN | Saudi Arabia | Biomedical Databases | SARS-CoV-2 | ANN | Modelling disease incidence in population | N/A | N/A | N/A |
| Hasan (2020) | A Methodological Approach for Predicting COVID-19 Epidemic Using EEMD-ANN Hybrid Model | Global | Biomedical Databases | SARS-CoV-2 | Hybrid model EEMD-ANN | Evaluating effectiveness of intervention | Model Implementation | N/A | N/A |
| Niazkar (2020) | Application of artificial neural networks to predict the COVID-19 outbreak | China, Japan, Singapore, Iran, Italy, South Africa and USA | Longitudinal Survey Data | SARS-CoV-2 | ANN | Modelling disease incidence in population | N/A | N/A | N/A |
| Mollalo (2020) | Artificial Neural Network Modeling of Novel Coronavirus (COVID-19) Incidence Rates across the Continental United States | USA | Database (USAfacts.org) | SARS-CoV-2 | MLP | Modelling disease incidence in population | N/A | N/A | N/A |
| Pourghasemi (2020) | Spatial modeling, risk mapping, change detection, and outbreak trend analysis of coronavirus (COVID-19) in Iran (days between February 19 and June 14, 2020) | Iran | Biomedical Databases | SARS-CoV-2 | RF | Modelling disease incidence in population AND modelling risk in population | N/A | N/A | N/A |
| Gupta (2020b) | Real-Time Analysis of COVID-19 Pandemic on Most Populated Countries Worldwide | USA, India, China, Pakistan, and Indonesia | Biomedical Databases | SARS-CoV-2 | Linear regression, SVR, and RF Regression | N/A | N/A | N/A |  |
| Behnood (2020) | Determinants of the infection rate of the COVID-19 in the U.S. using ANFIS and virus optimization algorithm (VOA) | USA | Longitudinal Survey Data | SARS-CoV-2 | ANFIS, VOA | Comparison of models/approaches | N/A | N/A | N/A |
| Al-Qaness (2020b) | Optimization Method for Forecasting Confirmed Cases of COVID-19 in China | China | Biomedical Databases | SARS-CoV-2 | ANFIS | Modelling disease incidence in population AND comparison of models/approaches | N/A | N/A | N/A |
| Al-qaness (2020a) | Marine Predators Algorithm for ForecastingConfirmed Cases of COVID-19 in Italy, USA,Iran and Korea | Italy, Iran, Korea, and the USA | Biomedical Databases | SARS-CoV-2 | ANFIS | Modelling disease incidence in population AND comparison of models/approaches | N/A | N/A | N/A |
| Al Mahmoud (2020) | Covid-19 Global Spread Analyzer: An ML-Based Attempt | Jordan | Biomedical Databases | SARS-CoV-2 | NaiveBayes, SMO, J48 and RF | Modelling disease incidence in population AND comparison of models/approaches | Model Design | N/A | N/A |
| Ahmad (2020) | Predictions of coronavirus COVID-19 distinct cases in Pakistan through an artificial neural network | Pakistan | Biomedical Databases | SARS-CoV-2 | ANN | Modelling disease incidence in population | N/A | N/A | N/A |
| Asfahan (2020) | Using a simple open-source automated machine learning algorithm to forecast COVID-19 spread: A modelling study | India | Biomedical Databases | SARS-CoV-2 | Linear and non-linear regression | Modelling disease incidence in population | N/A | N/A | N/A |
| Ghazaly (2020) | Novel Coronavirus Forecasting Model using Nonlinear Autoregressive Artificial Neural Network | Egypt, Saudia Arabia, Jordan, USA, Spain, Italy, France, Iran, Russian Federation | Biomedical Databases | SARS-CoV-2 | Non-Auto Regressive Neural Network | Modelling disease incidence in population | N/A | N/A | N/A |
| Saqib (2020) | Forecasting COVID-19 outbreak progression using hybrid polynomial-Bayesian ridge regression model | India | Biomedical Databases | SARS-CoV-2 | Polynomial-Bayesian ridge regression model | Modelling disease incidence in population | N/A | N/A | N/A |
| Ayyoubzadeh (2020) | Predicting COVID-19 Incidence Through Analysis of Google Trends Data in Iran: Data Mining and Deep Learning Pilot Study | Iran | Longitudinal Survey Data | SARS-CoV-2 | Linear regression, LSTM | Modelling disease incidence in population | N/A | N/A | N/A |
| Shahid (2020) | Predictions for COVID-19 with deep learning models of LSTM, GRU and Bi-LSTM | Pakistan | Biomedical Databases | SARS-CoV-2 | LSTM, bidirectional long short term memory (Bi-LSTM), GRU, SVR and ARIMA | Modelling disease incidence in population AND comparison of models/approaches | N/A | N/A | N/A |
| Rahmadani (2020) | Hybrid Deep Learning-Based Epidemic Prediction Framework of COVID-19: South Korea Case | South Korea | Biomedical Databases | SARS-CoV-2 | The proposed framework is the hybrid deep learning framework using the meta-population model and LSTM, and DNN | Modelling disease incidence in population AND comparison of models/approaches | Model Training | Estimation bias | incorporating a meta-population model with a deep learning approach. |
| Shastri (2020) | Time series forecasting of Covid-19 using deep learning models: India-USA comparative case study | India and USA | Longitudinal Survey Data | SARS-CoV-2 | Bi-directional LSTM, Convolutional LSTM , CoBiD ensemble (a type of RNN) | Comparison of models/approaches | N/A | N/A | N/A |
| Frauenfeld (2020) | Forecasting tuberculosis using diabetes-related google trends data | Germany | Google Trends (GTD) | Tuberculosis | SARIMA, autoregressive feed-forward neural network (NNAR) | N/A | N/A | N/A | N/A |
| Jia (2019) | Integrating Multiple Data Sources and Learning Models to Predict Infectious Diseases in China | China | Biomedical Databases | Diarrheal Diseases, Pertussis, Malaria, Tuberculosis | Linear model, ARIMA, boosting tree model (XGBoost), RNN (LSTM) | Modelling disease incidence in population | N/A | N/A | N/A |
| Abubakar (2019) | Computational intelligence-based model for diarrhea prediction using Demographic and Health Survey data | Nigeria | Biomedical Databases | Diarrheal Diseases | ANN | Modelling disease incidence in population | N/A | N/A | N/A |
| Li (2019) | Application of a hybrid model in predicting the incidence of tuberculosis in a Chinese population | China | Longitudinal Survey Data | Tuberculosis | ARIMA, RIMA-generalized regression neural network (GRNN) hybrid model. | Comparison of models/approaches | N/A | N/A | N/A |
| Fung (2017) | #Globalhealth Twitter Conversations on #Malaria, #HIV, #TB, #NCDS, and #NTDS: a Cross-Sectional Analysis | Global | Social Media | Malaria, HIV, noncommunicable diseases (NCDS), tuberculosis, and neglected tropical diseases (NTDS) | Latent Dirichlet Allocation analysis | Surveillance | N/A | N/A | N/A |
| Kapwata (2016) | Random forest variable selection in spatial malaria transmission modelling in Mpumalanga Province, South Africa | South Africa | Biomedical Databases | Malaria | RF | Surveillance AND modelling risk in population | N/A | N/A | N/A |
| Zhang (2015) | Effect of Meteorological Factors on Incidence of Tuberculosis: A 15-Year Retrospective Study Based on Chinese Medicine Theory of Five Circuits and Six Qi | China | Biomedical Databases | Tuberculosis | ANN | Modelling disease incidence in population | N/A | N/A | N/A |
| Buczak (2015) | Fuzzy association rule mining and classification for the prediction of malaria in South Korea | South Korea | Biomedical Databases | Malaria | Fuzzy association rule mining (FARM) | Modelling disease incidence in population | N/A | N/A | N/A |
| Atencia (2013) | Estimation of parameters based on artificial neural networks and threshold of HIV/AIDS epidemic system in Cuba | Spain | Biomedical Databases | HIV | Neural Network | Modelling disease incidence in population AND comparison of models/approaches | N/A | N/A | N/A |
| Kiang (2006) | Meteorological, environmental remote sensing and neural network analysis of the epidemiology of malaria transmission in Thailand | USA | parasitological, meteorological and environmental data | Malaria | Neural network | Evaluating effectiveness of intervention | N/A | N/A | N/A |
| Ture (2006) | Comparison of four different time series methods to forecast hepatitis A virus infection | Turkey | Biomedical Databases | Hepatitis A | ANN, MLP, radial basis function (RBF), and time delay neural networks (TDNN)), and ARIMA | Modelling disease incidence in population | N/A | N/A | N/A |
