# Supplementary material for: Machine Learning Used in Communicable Disease Control: A Scoping Review
Source: Public Health Rev. 2026 Feb 13;47:1608074. doi: 10.3389/phrs.2026.1608074 (PMC12945845; doi:10.3389/phrs.2026.1608074)
Supplement: Supplementary file 2 [file Supplementaryfile1.pdf]

Literature Search Strategies for: Machine Learning Models Related to Communicable Diseases in Population and Public Health: A Scoping Review

An information specialist (CZ) with Library Services, Unity Health Toronto carried out comprehensive searches using a combination of subject headings and keywords, adapted for each database, for the broad concepts of artificial intelligence combined with the following communicable diseases: lower respiratory infections, diarrheal diseases, tuberculosis, HIV, malaria, meningitis, measles, pertussis (whooping cough), hepatitis, SARS-CoV-2.
The following databases were searched from 2000 to the present on July, 14-15 2022: Medline (Ovid), Embase (Ovid), Cochrane Central Register of Controlled Trials and Cochrane Database of Systematic Reviews(Ovid), Scopus, ACM Digital Library, INSPEC, and Web of Science's Science Citation Index, Social Sciences Citation Index, and Emerging Sources Citation Index. All languages were included in the search. Commentaries, letters, editorials, conference proceedings were excluded. There were 47,310 results prior to de-duplication, with 28,457 results following deduplication in EndNote. Following an additional automatic deduplication in DistillerSR, a total of 28,378 records remained to be screened.

Ovid MEDLINE: Epub Ahead of Print, In-Process & Other Non-Indexed Citations, Ovid MEDLINE® Daily and Ovid MEDLINE® <1946-Present>

1	exp lung/ or pleura/ or trachea/	333007
2	Respiratory Tract Infections/	41679
3	1 and 22720	
4	Influenza, Human/	55409
5	exp Pneumonia/	271748
6	bronchitis/ or exp bronchiolitis/	29777
7	LRTI.tw,kf.	1490
8	lower respiratory tract infection*.tw,kf.	7497
9	LRTI.tw,kf.	1490
10	influenza.tw,kf.	109327
11	flu.tw,kf.	15470
12	exp influenzavirus a/ or exp influenzavirus b/ or influenzavirus c/	49586
13	pneumonia*.tw,kf.	199162
14	Bronchitis.tw,kf.	24187
15	bronchiolitis.tw,kf.	12409
16	pneumovirus/ or respiratory syncytial viruses/ or respiratory syncytial virus, human/	9784
17	Respiratory syncytial virus.tw,kf.	15215
18	(acute respiratory distress syndrome* or ARDS).tw,kf.	26949
19	exp Diarrhea/	56255
20	diarrhea*.tw,kf.	90102
21	diarrhoea*.tw,kf.	32190
22	Rotavirus Infections/	8518
23	rotavirus*.tw,kf.	16085
24	viral gastroenteritis.tw,kf.	1087

25 Campylobacter Infections/ 7660
26 Campylobacter.tw,kf. 17969
27 salmonella infections/ or salmonella food poisoning/ 16921
28 salmonella.tw,kf. 82005
29 Dysentery, Bacillary/ 8021
30 dysentery.tw,kf. 8685
31 Escherichia coli Infections/ 34559
32 (Escherichia coli or e coli).tw,kf. 340537
33 Clostridium Infections/ 10171
34 Clostridioides difficile/ 10857
35 (C diff* or Clostridioides difficile or Clostridium difficile or CDAD).tw,kf. 19261
36 exp Norovirus/ 5381
37 (norovirus* or norwalk virus*).tw,kf. 7126
38 exp Intestinal Diseases, Parasitic/ 27298
39 (Amebiasis or amoebiasis or entomoeba infection* or Entamoeba histolytica or E histolytica).tw,kf. 12303
40 (infectious colitis or bacterial colitis or CMV colitis or Cytomegalovirus colitis or amoebic colitis or viral colitis or Shigellosis or shigella or Yersinia enterocolitica or Pseudomembranous colitis).tw,kf. 24347
41 exp Tuberculosis/ 202475
42 Tuberculosis.tw,kf. 228808
43 TB.ti. 9115
44 TB.ab. 62704
45 exp HIV Infections/ 306903
46 (HIV or AIDS or human immunodeficiency virus or acquired immunodeficiency syndrome or acquired immuno-deficiency syndrome).tw,kf. 444552
47 exp Malaria/ 72193
48 malaria*.tw,kf. 93972
49 exp Measles/ or exp Measles virus/ 19847
50 measles.tw,kf. 25563
51 Whooping Cough/ 9001
52 (whooping cough or pertussis or pertusses).tw,kf. 30434
53 exp Hepatitis/ 178923
54 hepatitis.tw,kf.237777
55 exp Meningitis/ 57985
56 Meningitis.tw,kf. 58165
57 COVID-19/ or SARS-CoV-2/175516
58 (coronavirus/ or betacoronavirus/ or coronavirus infections/) and (disease outbreaks/ or epidemics/ or pandemics/) 40138
59 (nCoV* or 2019nCoV or 19nCoV or COVID19* or COVID or SARS-COV-2 or SARSCOV-2 or SARS-COV2 or SARSCOV2 or SARS coronavirus 2 or Severe Acute Respiratory Syndrome Coronavirus 2 or Severe Acute Respiratory Syndrome Corona Virus 2).ti,ab,kf,nm,ot,ox,rx,px. 264982
60 ((new or novel or "19" or "2019" or Wuhan or Hubei or China or Chinese) adj3 (coronavirus* or corona virus* or betacoronavirus* or CoV or HCoV)).ti,ab,kf,ot. 73165
61 (longCOVID* or postCOVID* or postcoronavirus* or postSARS*).ti,ab,kf,ot. 44

62 ((coronavirus* or corona virus* or betacoronavirus*) adj3 (pandemic* or epidemic* or outbreak* or crisis)).ti,ab,kf,ot. 12921
63 ((Wuhan or Hubei) adj5 pneumonia).ti,ab,kf,ot. 400
64 or/57-63 276564
65 limit 64 to yr="2019 -Current" [CADTH COVID-19 Medline Search filter https://covid-cadth-ca.myaccess.library.utoronto.ca/literature-searching-tools/cadth-covid-19-search-strings/#covid-19-medline ; modified by removing testing and vaccines] 275067
66 Communicable Diseases/ 32143
67 communicable disease*.tw,kf. not "non communicable disease*".m_titl. 18249
68 (or/3-56) or 65 or 66 or 67 [Communicable Diseases] 2427745
69 artificial intelligence/ or machine learning/ or deep learning/ or supervised machine learning/ or support vector machine/ or unsupervised machine learning/ 76421
70 computer heuristics/ or natural language processing/ or neural networks, computer/ 46383
71 artificial intelligence.tw,kf. 24336
72 Machine learning.tw,kf. 66493
73 neural network*.tw,kf. 79348
74 deep learning.tw,kf. 33063
75 supervised learning.tw,kf. 4231
76 unsupervised learning.tw,kf. 2108
77 deep architecture*.tw,kf. 273
78 computational intelligence.tw,kf. 425
79 computer reasoning.tw,kf. 7
80 machine intelligence.tw,kf. 228
81 support vector machine*.tw,kf. 21852
82 support vector network*.tw,kf. 7
83 natural language processing.tw,kf. 6054
84 data driven algorithm*.tw,kf. 154
85 perceptron.tw,kf. 3384
86 random forest*.tw,kf. 16359
87 (ensemble learning or reinforcement learning).tw,kf. 6397
88 AI.ti. 3657
89 or/69-88 [Machine Learning] 218134
90 68 and 89 11482
91 limit 90 to (comment or editorial or letter) 217
92 90 not 91 11265
93 92 not (exp animals/ not humans.sh.) 11008
94 limit 93 to yr="2000 -Current" 10801

EBM Reviews - Cochrane Central Register of Controlled Trials <June 2022>
EBM Reviews - Cochrane Database of Systematic Reviews <2005 to July 13, 2022>

1 exp lung/ or pleura/ or trachea/ 5090
2 Respiratory Tract Infections/ 2453

3 1 and 247
4 Influenza, Human/ 2950
5 exp Pneumonia/ 5921
6 bronchitis/ or exp bronchiolitis/ 1786
7 LRTI.tw,kf. 395
8 lower respiratory tract infection*.tw,kf. 1352
9 LRTI.tw,kf. 395
10 influenza.tw,kf. 7470
11 flu.tw,kf. 2291
12 exp influenzavirus a/ or exp influenzavirus b/ or influenzavirus c/ 999
13 pneumonia*.tw,kf. 16706
14 Bronchitis.tw,kf. 3226
15 bronchiolitis.tw,kf. 1580
16 pneumovirus/ or respiratory syncytial viruses/ or respiratory syncytial virus, human/ 188
17 Respiratory syncytial virus.tw,kf. 1029
18 (acute respiratory distress syndrome* or ARDS).tw,kf. 3247
19 exp Diarrhea/ 3673
20 diarrhea*.tw,kf. 15615
21 diarrhoea*.tw,kf. 6684
22 Rotavirus Infections/ 392
23 rotavirus*.tw,kf. 1033
24 viral gastroenteritis.tw,kf. 55
25 Campylobacter Infections/ 70
26 Campylobacter.tw,kf. 330
27 salmonella infections/ or salmonella food poisoning/ 60
28 salmonella.tw,kf. 681
29 Dysentery, Bacillary/ 185
30 dysentery.tw,kf. 223
31 Escherichia coli Infections/ 414
32 (Escherichia coli or e coli).tw,kf. 3107
33 Clostridium Infections/ 187
34 Clostridioides difficile/ 200
35 (C diff* or Clostridioides difficile or Clostridium difficile or CDAD).tw,kf. 1796
36 exp Norovirus/ 34
37 (norovirus* or norwalk virus*).tw,kf. 163
38 exp Intestinal Diseases, Parasitic/ 287
39 (Amebiasis or amoebiasis or entomoeba infection* or Entamoeba histolytica or E histolytica).tw,kf. 170
40 (infectious colitis or bacterial colitis or CMV colitis or Cytomegalovirus colitis or amoebic colitis or viral colitis or Shigellosis or shigella or Yersinia enterocolitica or Pseudomembranous colitis).tw,kf. 549
41 exp Tuberculosis/ 2607
42 Tuberculosis.tw,kf. 6887
43 TB.ti. 842
44 TB.ab. 3797

45 exp HIV Infections/ 13588
46 (HIV or AIDS or human immunodeficiency virus or acquired immunodeficiency syndrome or acquired immuno-deficiency syndrome).tw,kf. 35709
47 exp Malaria/ 3370
48 malaria*.tw,kf. 7307
49 exp Measles/ or exp Measles virus/ 341
50 measles.tw,kf. 1285
51 Whooping Cough/ 351
52 (whooping cough or pertussis or pertusses).tw,kf. 1843
53 exp Hepatitis/ 6955
54 hepatitis.tw,kf. 20951
55 exp Meningitis/ 701
56 Meningitis.tw,kf. 1945
57 COVID-19/ or SARS-CoV-2/ 1958
58 (coronavirus/ or betacoronavirus/ or coronavirus infections/) and (disease outbreaks/ or epidemics/ or pandemics/) 132
59 ((new or novel or "19" or "2019" or Wuhan or Hubei or China or Chinese) adj3 (coronavirus* or corona virus* or betacoronavirus* or CoV or HCoV)).ti,ab,kf,ot. 2900
60 (nCoV* or 2019nCoV or 19nCoV or COVID19* or COVID or SARS-COV-2 or SARSCOV-2 or SARS-COV2 or SARSCOV2 or SARS coronavirus 2 or Severe Acute Respiratory Syndrome Coronavirus 2 or Severe Acute Respiratory Syndrome Corona Virus 2).ti,ab,kf. 11478
61 (longCOVID* or postCOVID* or postcoronavirus* or postSARS*).ti,ab,kf,ot. 9
62 ((coronavirus* or corona virus* or betacoronavirus*) adj3 (pandemic* or epidemic* or outbreak* or crisis)).ti,ab,kf,ot. 260
63 ((Wuhan or Hubei) adj5 pneumonia).ti,ab,kf,ot. 25
64 or/57-63 11691
65 limit 64 to yr="2019 -Current" 11611
66 Communicable Diseases/ 2223
67 communicable disease*.tw,kf. not "non communicable disease*".m_titl. 785
68 (or/3-56) or 65 or 66 or 67 [Communicable Diseases] 138593
69 artificial intelligence/ or machine learning/ or deep learning/ or supervised machine learning/ or support vector machine/ or unsupervised machine learning/ 502
70 computer heuristics/ or natural language processing/ or neural networks, computer/ 156
71 artificial intelligence.tw,kf. 886
72 Machine learning.tw,kf. 1963
73 neural network*.tw,kf. 1659
74 deep learning.tw,kf. 747
75 supervised learning.tw,kf. 50
76 unsupervised learning.tw,kf. 18
77 deep architecture*.tw,kf. 1
78 computational intelligence.tw,kf. 5
79 computer reasoning.tw,kf. 0
80 machine intelligence.tw,kf. 8
81 support vector machine*.tw,kf. 466

82 support vector network*.tw,kf. 0
83 natural language processing.tw,kf. 197
84 data driven algorithm*.tw,kf. 7
85 perceptron.tw,kf. 65
86 random forest*.tw,kf. 633
87 (ensemble learning or reinforcement learning).tw,kf. 195
88 AI.ti. 353
89 or/69-88 [Machine Learning] 5504
90 68 and 89 314
91 limit 90 to yr="2000 -Current" 265
92 remove duplicates from 91 262

Embase Classic+Embase <1947 to 2022 July 14> (Ovid)

1 exp lower respiratory tract infection/ 297297
2 exp influenza/ 109299
3 exp pneumonia/ 387906
4 exp bronchitis/77075
5 pneumovirus infection/ or respiratory syncytial virus infection/ 6882
6 exp diarrhea/ 304707
7 exp Rotavirus infection/ 4548
8 viral gastroenteritis/ 2642
9 exp campylobacteriosis/ 3154
10 exp salmonellosis/ 37200
11 exp dysentery/ 15350
12 exp Escherichia coli infection/ 10853
13 exp Clostridium infection/ 46985
14 exp norovirus infection/ 1726
15 exp intestine infection/ 90131
16 exp tuberculosis/ 285711
17 exp Human immunodeficiency virus infection/ 409464
18 exp Human immunodeficiency virus infected patient/ 47799
19 exp malaria/ 109147
20 exp measles/ 28170
21 pertussis/ 17889
22 exp hepatitis/ 362938
23 exp meningitis/ 133953
24 communicable disease/ 36097
25 (LRTI or lower respiratory tract infection* or LRTI or influenza or flu or pneumonia* or Bronchitis or bronchiolitis or Respiratory syncytial virus or acute respiratory distress syndrome* or ARDS or diarrhea* or diarrhoea* or rotavirus* or viral gastroenteritis or Campylobacter or salmonella or dysentery or Escherichia coli or e coli or C diff* or Clostridioides difficile or Clostridium difficile or CDAD or norovirus* or norwalk virus* or Amebiasis or amoebiasis or entomoeba infection* or Entamoeba histolytica or E histolytica or infectious colitis or bacterial colitis or CMV colitis or Cytomegalovirus colitis or amoebic colitis or viral colitis or Shigellosis

or shigella or Yersinia enterocolitica or Pseudomembranous colitis or HIV or AIDS or human immunodeficiency virus or acquired immunodeficiency syndrome or acquired immuno-deficiency syndrome or Tuberculosis or TB or malaria* or measles or whooping cough or pertussis or pertusses or hepatitis or Meningitis).tw,kf. 2497914
26 communicable disease*.tw,kf. not "non communicable disease*".m_titl. 18278
27 1 or 2 or 3 or 4 or 5 or 6 or 7 or 8 or 9 or 10 or 11 or 12 or 13 or 14 or 15 or 16 or 17 or 18 or 19 or 20 or 21 or 22 or 23 or 24 or 25 or 26 3244986
28 exp coronavirus disease 2019/ 241439
29 (coronavirinae/ or betacoronavirus/ or coronavirus infection/) and (epidemic/ or pandemic/) 9296
30 (nCoV* or 2019nCoV or 19nCoV or COVID19* or COVID or SARS-COV-2 or SARSCOV-2 or SARS-COV2 or SARSCOV2 or SARS coronavirus 2 or Severe Acute Respiratory Syndrome Coronavirus 2 or Severe Acute Respiratory Syndrome Corona Virus 2).tw,kf. 285566
31 ((new or novel or "19" or "2019" or Wuhan or Hubei or China or Chinese) adj3 (coronavirus* or corona virus* or betacoronavirus* or CoV or HCoV)).tw,kf. 74033
32 ((coronavirus* or corona virus* or betacoronavirus*) adj3 (pandemic* or epidemic* or outbreak* or crisis)).tw,kf. 12775
33 or/29-32 [Revised terms from CADTH COVID-19 Embase filter last edited Sept 2, 2021 https://covid.cadth.ca/literature-searching-tools/cadth-covid-19-search-strings/] 292331
34 limit 33 to yr="2020 -Current" 290889
35 27 or 28 or 34 3489586
36 exp machine learning/ 320806
37 exp artificial intelligence/ 63390
38 natural language processing/ 8104
39 (artificial intelligence or Machine learning or neural network* or deep learning or supervised learning or unsupervised learning or deep architecture* or computational intelligence or computer reasoning or machine intelligence or support vector machine* or support vector network* or natural language processing or data driven algorithm* or perceptron or random forest* or ensemble learning or reinforcement learning).tw,kf. 224802
40 AI.m_titl. 4865
41 36 or 37 or 38 or 39 or 40 406445
42 35 and 41 28323
43 limit 42 to (chapter or conference abstract or conference paper or "conference review" or editorial or letter) 3779
44 42 not 43 24544
45 44 not ((exp animal/ or animal experiment/ or nonhuman/) not (exp human/ or human experiment/)) 18084
46 limit 45 to yr="2000 -Current" 17915
47 limit 46 to embase 13950

EBM Reviews - Cochrane Central Register of Controlled Trials <January 2022>
EBM Reviews - Cochrane Database of Systematic Reviews <2005 to March 2, 2022> (Ovid)

1 artificial intelligence/ or machine learning/ or deep learning/ or supervised machine learning/ or support vector machine/ or unsupervised machine learning/ 400
2 computer heuristics/ or natural language processing/ or neural networks, computer/ 138
3 (artificial intelligence or Machine learning or neural network* or deep learning or supervised learning or unsupervised learning or deep architecture* or computational intelligence or computer reasoning or machine intelligence or support vector machine* or support vector network* or natural language processing or data driven algorithm* or perceptron or random forest* or ensemble learning or reinforcement learning).tw. 4740
4 1 or 2 or 3 4892
5 exp Respiratory Tract Neoplasms/ 8880
6 (lung* adj3 (cancer* or neoplas* or adenocarcinoma* or carcinoma* or malignan* or tumour* or tumor* or blastoma* or metast*)).tw. 23875
7 (pulmonary adj3 (cancer* or neoplas* or adenocarcinoma* or carcinoma* or malignan* or tumour* or tumor* or blastoma* or metast*)).tw. 1123
8 (bronch* adj3 (cancer* or neoplas* or adenocarcinoma* or carcinoma* or malignan* or tumour* or tumor* or blastoma* or metast*)).tw. 946
9 (alveolar adj3 (cancer* or neoplas* or adenocarcinoma* or carcinoma* or malignan* or tumour* or tumor* or blastoma* or metast*)).tw. 33
10 (trachea* adj3 (cancer* or neoplas* or adenocarcinoma* or carcinoma* or malignan* or tumour* or tumor* or blastoma* or metast*)).tw. 47
11 (pleural adj3 (cancer* or neoplas* or adenocarcinoma* or carcinoma* or malignan* or tumour* or tumor* or blastoma* or metast*)).tw. 1286
12 (Malignant Mesothelioma or Multiple Pulmonary Nodules or Pancoast Syndrome or pancoast tumor* or pancoast tumour*).tw. 177
13 exp Myocardial Ischemia/ 29090
14 (Myocardial Ischemia* or Myocardial Ischaemia or ischemic heart disease* or ischaemic heart disease* or Acute Coronary Syndrome* or angina* or angor pectori* or myocardial preinfarction syndrome* or stenocardia* or coronary artery insufficiency or coronary artery occlusive disease* or coronary heart disease* or coronary insufficiency or coronary occlusive disease* or ischaemic cardiac disease* or ischaemic cardial disease* or ischaemic cardiopathy or ischemic cardiac disease* or ischemic cardial disease* or ischemic cardiopathy or Coronary Disease* or Coronary Aneurysm* or Coronary Artery Disease* or Coronary Occlusion or Coronary Stenosis or Coronary Restenosis or Coronary Thrombosis or Coronary Vasospasm or Myocardial Infarct* or stemi or cardiovascular stroke* or heart attack*).tw. 64219
15 Diabetes Mellitus, Type 2/ 19442
16 (type* adj3 two adj3 diabet*).tw. 456
17 (type* adj3 "2" adj3 diabet*).tw. 41555
18 (type* adj3 "II" adj3 diabet*).tw. 2995
19 (adult* adj3 onset adj3 diabet*).tw. 111
20 (Matur* adj3 onset adj3 diabet*).tw. 98
21 (slow adj3 onset adj3 diabet*).tw. 2
22 (Ketosis resistant adj3 diabet*).tw. 1
23 (stable adj3 diabet*).tw. 635
24 (Non insulin adj3 dependent adj3 diabet*).tw. 2130
25 (Noninsulin adj3 dependent adj3 diabet*).tw. 152

26 NIDDM.tw. 1110
27 MODY.tw. 57
28 "diabet*".m_titl. 62872
29 exp Pulmonary Disease, Chronic Obstructive/ 6146
30 (chronic obstructive lung disease or chronic airflow obstruction or chronic airway obstruction or chronic obstructive bronchopulmonary disease or chronic obstructive lung disorder or chronic obstructive pulmonary disease or chronic obstructive pulmonary disorder or chronic obstructive respiratory disease or chronic pulmonary obstructive disease or chronic pulmonary obstructive disorder or copd or lung chronic obstructive disease or obstructive chronic lung disease or obstructive chronic pulmonary disease or chronic bronchitis or emphysema).tw. 24277
31 exp Dementia/ 6445
32 (Dementia* or demented or amentia* or alzheimer* or Primary Progressive aphasia or mesulam syndrome or Creutzfeldt Jakob Syndrome or creutzfeldt jakob disease or CADASIL or Frontotemporal Lobar Degeneration or pick* disease or lobar atroph* or Huntington* Disease or huntington* chorea or Kluver Bucy Syndrome or Lewy Body Disease* or Lewy Body Disorder*).tw. 23352
33 or/5-32 212821
34 4 and 33 629
35 remove duplicates from 34 616
36 limit 35 to yr="2000 -Current" 578

Search History
Interface - EBSCOhost Research Databases
Search Screen - Advanced Search
Database - **CINAHL Complete**

#	Query	Limiters/Expanders	Results
S38	S37 NOT ((((MH "Animals+") OR (MH "Animal Studies") OR (TI "animal model*")) NOT (MH "human")))	Limiters - Published Date: 20000101-20231231<br>Expanders - Apply equivalent subjects<br>Search modes - Boolean/Phrase	1,895
S37	S35 NOT S36	Expanders - Apply equivalent subjects<br>Search modes - Boolean/Phrase	1,922
S36	S35	Limiters - Publication Type: Book, Book	108

		Chapter, Book Review, Commentary, Doctoral Dissertation, Editorial, Letter, Masters Thesis, Response<br>Expanders - Apply equivalent subjects<br>Search modes - Boolean/Phrase	
S35	S4 AND S34	Expanders - Apply equivalent subjects<br>Search modes - Boolean/Phrase	2,030
S34	S5 OR S6 OR S7 OR S8 OR S9 OR S10 OR S11 OR S12 OR S13 OR S14 OR S15 OR S16 OR S17 OR S18 OR S19 OR S20 OR S21 OR S22 OR S23 OR S24 OR S25 OR S26 OR S27 OR S28 OR S29 OR S33	Expanders - Apply equivalent subjects<br>Search modes - Boolean/Phrase	532,580
S33	S30 OR S31 OR S32	Limiters - Published Date: 20200101-20221231<br>Expanders - Apply equivalent subjects<br>Search modes - Boolean/Phrase	105,528
S32	((coronavirus* or corona virus* or betacoronavirus*) N3 (pandemic* or epidemic* or outbreak* or crisis))	Expanders - Apply equivalent subjects<br>Search modes - Boolean/Phrase	17,624
S31	((new or novel or "19" or "2019" or Wuhan or Hubei or China or Chinese) N3 (coronavirus* or corona virus* or betacoronavirus* or CoV or HCoV))	Expanders - Apply equivalent subjects<br>Search modes - Boolean/Phrase	18,264
S30	nCoV* or 2019nCoV or 19nCoV or COVID19* or COVID or SARS-COV-2 or SARSCOV-2 or SARS-COV2 or SARSCOV2 or SARS coronavirus 2 or Severe Acute Respiratory Syndrome Coronavirus 2 or Severe Acute Respiratory Syndrome Corona Virus 2)	Expanders - Apply equivalent subjects<br>Search modes - Boolean/Phrase	103,820
S29	communicable disease* NOT non communicable disease*	Expanders - Apply equivalent subjects	13,592

		Search modes - Boolean/Phrase	
S28	(LRTI or lower respiratory tract infection* or LRTI or influenza or flu or pneumonia* or Bronchitis or bronchiolitis or Respiratory syncytial virus or acute respiratory distress syndrome* or ARDS or diarrhea* or diarrhoea* or rotavirus* or viral gastroenteritis or Campylobacter or salmonella or dysentery or Escherichia coli or e coli or C diff* or Clostridioides difficile or Clostridium difficile or CDAD or norovirus* or norwalk virus* or Amebiasis or amoebiasis or entomoeba infection* or Entamoeba histolytica or E histolytica or infectious colitis or bacterial colitis or CMV colitis or Cytomegalovirus colitis or amoebic colitis or viral colitis or Shigellosis or shigella or Yersinia enterocolitica or Pseudomembranous colitis or HIV or AIDS or human immunodeficiency virus or acquired immunodeficiency syndrome or acquired immuno-deficiency syndrome or Tuberculosis or TB or malaria* or measles or whooping cough or pertussis or pertusses or hepatitis or Meningitis)	Expanders - Apply equivalent subjects Search modes - Boolean/Phrase	398,914
S27	(MH "COVID-19+")	Expanders - Apply equivalent subjects Search modes - Boolean/Phrase	34,297
S26	(MH "Communicable Diseases")	Expanders - Apply equivalent subjects Search modes - Boolean/Phrase	12,718
S25	(MH "Hepatitis+")	Expanders - Apply equivalent subjects Search modes - Boolean/Phrase	31,086
S24	(MH "HIV Infections+") OR (MH "HIV-Positive Persons+")	Expanders - Apply equivalent subjects Search modes - Boolean/Phrase	103,876
S23	(MH "Malaria")	Expanders - Apply equivalent subjects	9,600

		Search modes - Boolean/Phrase	
S22	(MH "Meningitis+")	Expanders - Apply equivalent subjects Search modes - Boolean/Phrase	7,827
S21	(MH "Whooping Cough")	Expanders - Apply equivalent subjects Search modes - Boolean/Phrase	2,163
S20	(MH "Measles+")	Expanders - Apply equivalent subjects Search modes - Boolean/Phrase	3,627
S19	(MH "Tuberculosis+")	Expanders - Apply equivalent subjects Search modes - Boolean/Phrase	25,420
S18	(MH "Caliciviridae Infections")	Expanders - Apply equivalent subjects Search modes - Boolean/Phrase	1,005
S17	(MH "Clostridium Infections+")	Expanders - Apply equivalent subjects Search modes - Boolean/Phrase	9,182
S16	(MH "Salmonella Infections") OR (MH "Escherichia Coli Infections")	Expanders - Apply equivalent subjects Search modes - Boolean/Phrase	5,178
S15	(MH "Dysentery+")	Expanders - Apply equivalent subjects Search modes - Boolean/Phrase	685
S14	(MH "Campylobacter Infections")	Expanders - Apply equivalent subjects Search modes - Boolean/Phrase	865

S13	(MH "Rotavirus Infections")	Expanders - Apply equivalent subjects Search modes - Boolean/Phrase	1,605
S12	(MH "Diarrhea")	Expanders - Apply equivalent subjects Search modes - Boolean/Phrase	11,878
S11	(MH "Respiratory Syncytial Virus Infections")	Expanders - Apply equivalent subjects Search modes - Boolean/Phrase	2,185
S10	(MH "Bronchitis+") OR (MH "Bronchopneumonia")	Expanders - Apply equivalent subjects Search modes - Boolean/Phrase	4,682
S9	(MH "Pneumonia+")	Expanders - Apply equivalent subjects Search modes - Boolean/Phrase	33,119
S8	(MH "Influenza+")	Expanders - Apply equivalent subjects Search modes - Boolean/Phrase	21,560
S7	S5 AND S6	Expanders - Apply equivalent subjects Search modes - Boolean/Phrase	287
S6	(MH "Lung+") OR (MH "Pleura") OR (MH "Trachea")	Expanders - Apply equivalent subjects Search modes - Boolean/Phrase	29,943
S5	(MH "Respiratory Tract Infections")	Expanders - Apply equivalent subjects Search modes - Boolean/Phrase	9,397
S4	S1 OR S2 OR S3	Expanders - Apply equivalent subjects Search modes - Boolean/Phrase	32,412

S3	TI AI	Expanders - Apply equivalent subjects Search modes - Boolean/Phrase	1,795
S2	(artificial intelligence or Machine learning or neural network* or deep learning or supervised learning or unsupervised learning or deep architecture* or computational intelligence or computer reasoning or machine intelligence or support vector machine* or support vector network* or natural language processing or data driven algorithm* or perceptron or random forest* or ensemble learning or reinforcement learning)	Expanders - Apply equivalent subjects Search modes - Boolean/Phrase	31,205
S1	(MH "Artificial Intelligence") OR (MH "Expert Systems") OR (MH "Knowbots") OR (MH "Machine Learning+") OR (MH "Natural Language Processing") OR (MH "Neural Networks (Computer)")	Expanders - Apply equivalent subjects Search modes - Boolean/Phrase	15,560

Saved at SMH zieglerc/library

Scopus
5,831 document results
(TITLE-ABS-KEY (("artificial intelligence" OR "Machine learning" OR "neural network*" OR "deep learning" OR "supervised learning" OR "unsupervised learning" OR "deep architecture*" OR "computational intelligence" OR "computer reasoning" OR "machine intelligence" OR "support vector machine*" OR "support vector network*" OR "natural language processing" OR "data driven algorithm* " OR perceptron OR "random forest*" OR "ensemble learning" OR "reinforcement learning"))) AND ((TITLE-ABS-KEY ((lrti OR "lower respiratory tract infection*" OR lrti OR influenza OR flu OR pneumonia* OR bronchitis OR bronchiolitis OR "Respiratory syncytial virus" OR "acute respiratory distress syndrome*" OR ards OR diarrhea* OR diarrhoea* OR rotavirus* OR "viral gastroenteritis" OR campylobacter OR salmonella OR dysentery OR "Escherichia coli" OR "e coli" OR "C diff*" OR "Clostridioides difficile" OR "Clostridium difficile" OR norovirus* OR "norwalk virus*" OR amebiasis OR amoebiasis OR "entomoeba infection*" OR "Entamoeba histolytica" OR "E histolytica" OR "infectious colitis" OR "bacterial colitis" OR "CMV colitis" OR "Cytomegalovirus colitis" OR "amoebic colitis" OR "viral colitis" OR shigellosis OR shigella OR "Yersinia enterocolitica" OR "Pseudomembranous colitis" OR hiv OR aids OR "human immunodeficiency virus" OR "acquired immunodeficiency syndrome" OR "acquired immuno-deficiency syndrome" OR tuberculosis OR tb OR malaria* OR measles OR whooping AND cough OR pertussis OR pertusses OR hepatitis OR meningitis))) OR (TITLE-ABS-KEY (coronavirus* W/3 (pandemic* OR epidemic* OR outbreak* OR crisis))) OR ((TITLE ("communicable disease*") AND NOT TITLE ("non communicable disease*"))) OR (TITLE-ABS-KEY ((ncov* OR

2019ncov OR 19ncov OR covid19* OR covid OR "SARS-COV-2" OR "SARSCOV-2" OR "SARS-COV2" OR sarscov2 OR "SARS coronavirus 2" OR "Severe Acute Respiratory Syndrome Coronavirus 2" OR "Severe Acute Respiratory Syndrome Corona Virus 2")))) AND NOT (TITLE (animal* OR nonhuman* OR veterinar* OR avian* OR baboon* OR bird* OR bovine OR canine OR cat OR cats OR cattle* OR chick* OR chimp* OR cow OR cows OR dog OR dogs OR duck OR feline OR fish* OR geese OR goose OR macaque* OR marmoset* OR mice OR mouse OR murine OR ovine OR pig OR pigs OR piglet* OR porcine OR primate* OR rabbit OR rat OR rats OR rodent* OR sheep OR swine OR trout* OR zebrafish*) AND NOT (human* OR patient* OR women OR woman OR men OR man)) AND NOT INDEX (medline) AND (EXCLUDE (DOCTYPE , "cp") OR EXCLUDE (DOCTYPE , "cr") OR EXCLUDE (DOCTYPE , "ed") OR EXCLUDE (DOCTYPE , "ch") OR EXCLUDE (DOCTYPE , "le") OR EXCLUDE (DOCTYPE , "bk")) AND (LIMIT-TO (PUBYEAR , 2022) OR LIMIT-TO (PUBYEAR , 2021) OR LIMIT-TO (PUBYEAR , 2020) OR LIMIT-TO (PUBYEAR , 2019) OR LIMIT-TO (PUBYEAR , 2018) OR LIMIT-TO (PUBYEAR , 2017) OR LIMIT-TO (PUBYEAR , 2016) OR LIMIT-TO (PUBYEAR , 2015) OR LIMIT-TO (PUBYEAR , 2014) OR LIMIT-TO (PUBYEAR , 2013) OR LIMIT-TO (PUBYEAR , 2012) OR LIMIT-TO (PUBYEAR , 2011) OR LIMIT-TO (PUBYEAR , 2010) OR LIMIT-TO (PUBYEAR , 2009) OR LIMIT-TO (PUBYEAR , 2008) OR LIMIT-TO (PUBYEAR , 2007) OR LIMIT-TO (PUBYEAR , 2006) OR LIMIT-TO (PUBYEAR , 2005) OR LIMIT-TO (PUBYEAR , 2004) OR LIMIT-TO (PUBYEAR , 2003) OR LIMIT-TO (PUBYEAR , 2002) OR LIMIT-TO (PUBYEAR , 2001) OR LIMIT-TO (PUBYEAR , 2000))

Web of Science

Editions = ESCI , SCI-EXPANDED , SSCI

Query	Search History	Results
1	((TI=(("artificial intelligence" OR "Machine learning" OR "neural network*" OR "deep learning" OR "supervised learning" OR "unsupervised learning" OR "deep architecture*" OR "computational intelligence" OR "computer reasoning" OR "machine intelligence" OR "support vector machine*" OR "support vector network*" OR "natural language processing" OR "data driven algorithm* " OR perceptron OR "random forest*" OR "ensemble learning" OR "reinforcement learning"))) OR AB=(("artificial intelligence" OR "Machine learning" OR "neural network*" OR "deep learning" OR "supervised learning" OR "unsupervised learning" OR "deep architecture*" OR "computational intelligence" OR "computer reasoning" OR "machine intelligence" OR "support vector machine*" OR "support vector network*" OR "natural language processing" OR "data driven algorithm* " OR perceptron OR "random forest*" OR "ensemble learning" OR "reinforcement learning"))) OR TI=(AI)<br><br>Timespan: 2000-01-01 to 2023-12-31 (Publication Date)	517,355

2	TI=((LRTI or "lower respiratory tract infection*" or LRTI or influenza or flu or pneumonia* or Bronchitis or bronchiolitis or "Respiratory syncytial virus" or "acute respiratory distress syndrome*" or ARDS or diarrhea* or diarrhoea* or rotavirus* or "viral gastroenteritis" or Campylobacter or salmonella or dysentery or "Escherichia coli" or "e coli" or "C diff*" or "Clostridioides difficile" or "Clostridium difficile" or norovirus* or "norwalk virus*" or Amebiasis or amoebiasis or "entomoeba infection*" or "Entamoeba histolytica" or "E histolytica" or "infectious colitis" or "bacterial colitis" or "CMV colitis" or "Cytomegalovirus colitis" or "amoebic colitis" or "viral colitis" or Shigellosis or shigella or "Yersinia enterocolitica" or "Pseudomembranous colitis" or HIV or AIDS or "human immunodeficiency virus" or "acquired immunodeficiency syndrome" or "acquired immuno-deficiency syndrome" or Tuberculosis or TB or malaria* or measles or "whooping cough" or pertussis or pertusses or hepatitis or Meningitis))<br><br>Timespan: 2000-01-01 to 2023-12-31 (Publication Date)	860,976
3	(TI=((nCoV* or 2019nCoV or 19nCoV or COVID19* or COVID or "SARS-COV-2" or "SARSCOV-2" or "SARS-COV2" or SARSCOV2 or "SARS coronavirus 2" or "Severe Acute Respiratory Syndrome Coronavirus 2" or "Severe Acute Respiratory Syndrome Corona Virus 2"))) OR AB=((nCoV* or 2019nCoV or 19nCoV or COVID19* or COVID or "SARS-COV-2" or "SARSCOV-2" or "SARS-COV2" or SARSCOV2 or "SARS coronavirus 2" or "Severe Acute Respiratory Syndrome Coronavirus 2" or "Severe Acute Respiratory Syndrome Corona Virus 2"))<br><br>Timespan: 2000-01-01 to 2023-12-31 (Publication Date)	304,566
4	#3 OR #2	1,155,200
5	#1 AND #4	11,821
6	TI=((animal* OR nonhuman* OR veterinar* OR avian* OR baboon* OR bird* OR bovine OR canine OR cat OR cats OR cattle* OR chick* OR chimp* OR cow OR cows OR dog OR dogs OR duck OR feline OR fish* OR geese OR goose OR macaque* OR marmoset* OR mice OR mouse OR murine OR ovine OR pig OR pigs OR piglet* OR porcine OR primate* OR rabbit OR rat OR rats OR rodent* OR sheep OR swine OR trout* OR zebrafish*))	3,721,211
7	#5 NOT #6	11,666
8	(#5) NOT #6 and News Items or Book Reviews or Book Chapters or Letters or Proceedings Papers or Meeting Abstracts or Editorial Materials (Exclude – Document Types)	10,933

ACM Digital Library (Association for Computing Machinery)

78 Results for: [[Abstract: "artificial intelligence"] OR [Abstract: "machine learning"] OR [Abstract: "neural network"] OR [Abstract: "deep learning"] OR [Abstract: "supervised learning"] OR [Abstract: "unsupervised learning"] OR [Abstract: "deep architecture"] OR [Abstract: "computational intelligence"] OR [Abstract: "computer reasoning"] OR [Abstract: "machine intelligence"] OR [Abstract: "support vector machine"] OR [Abstract: "support vector network"] OR [Abstract: "natural language processing"] OR [Abstract: "data driven algorithm"] OR [Abstract: perceptron] OR [Abstract: "random forest"] OR [Abstract: "ensemble learning"] OR [Abstract: "reinforcement learning"]] AND [[Abstract: lrti or] OR [Abstract: "lower respiratory tract infection"] OR [Abstract: or influenza or flu or pneumonia or bronchitis or bronchiolitis or diarrhea* or diarrhoea* or] OR [Abstract: "viral gastroenteritis"] OR [Abstract: or campylobacter or salmonella or dysentery or] OR [Abstract: "escherichia coli"] OR [Abstract: or] OR [Abstract: "e coli"] OR [Abstract: or] OR [Abstract: "c difficile"] OR [Abstract: or] OR [Abstract: "clostridium difficile"] OR [Abstract: or norovirus* or] OR [Abstract: "norwalk virus"] OR [Abstract: or] OR [Abstract: "infectious colitis"] OR [Abstract: or] OR [Abstract: "bacterial colitis"] OR [Abstract: or] OR [Abstract: "cmv colitis"] OR [Abstract: or] OR [Abstract: "cytomegalovirus colitis"] OR [Abstract: or] OR [Abstract: "amoebic colitis"] OR [Abstract: or] OR [Abstract: "viral colitis"] OR [Abstract: or shigellosis or shigella or hiv or aids or] OR [Abstract: "human immunodeficiency virus"] OR [Abstract: or] OR [Abstract: "acquired immunodeficiency syndrome"] OR [Abstract: or] OR [Abstract: "acquired immuno-deficiency syndrome"] OR [Abstract: or tuberculosis or tb or malaria* or measles or] OR [Abstract: "whooping cough"] OR [Abstract: or pertussis or pertusses or hepatitis or meningitis] OR [Abstract: ncov* or 2019ncov or 19ncov or covid19* or covid or "sars-cov-2" or "sarscov-2" or "sars-cov2" or sarscov2 or] OR [Abstract: "sars coronavirus 2"] OR [Abstract: or] OR [Abstract: "severe acute respiratory syndrome coronavirus 2"] OR [Abstract: or] OR [Abstract: "severe acute respiratory syndrome corona virus 2"]] AND [Publication Date: (01/01/2000 TO 31/12/2022)]

Limited to Journals

4 Results for: [[Title: "artificial intelligence"] OR [Title: "machine learning"] OR [Title: "neural network"] OR [Title: "deep learning"] OR [Title: "supervised learning"] OR [Title: "unsupervised learning"] OR [Title: "deep architecture"] OR [Title: "computational intelligence"] OR [Title: "computer reasoning"] OR [Title: "machine intelligence"] OR [Title: "support vector machine"] OR [Title: "support vector network"] OR [Title: "natural language processing"] OR [Title: "data driven algorithm"] OR [Title: perceptron] OR [Title: "random forest"] OR [Title: "ensemble learning"] OR [Title: "reinforcement learning"]] AND [[Title: lrti or] OR [Title: "lower respiratory tract infection"] OR [Title: or influenza or flu or pneumonia or bronchitis or bronchiolitis or diarrhea* or diarrhoea* or] OR [Title: "viral gastroenteritis"] OR [Title: or campylobacter or salmonella or dysentery or] OR [Title: "escherichia coli"] OR [Title: or] OR [Title: "e coli"] OR [Title: or] OR [Title: "c difficile"] OR [Title: or] OR [Title: "clostridium difficile"] OR [Title: or norovirus* or] OR [Title: "norwalk virus"] OR [Title: or] OR [Title: "infectious colitis"] OR [Title: or] OR [Title: "bacterial colitis"] OR [Title: or] OR [Title: "cmv colitis"] OR [Title: or] OR [Title: "cytomegalovirus colitis"] OR [Title: or] OR [Title: "amoebic colitis"] OR [Title: or] OR [Title: "viral colitis"] OR [Title: or shigellosis or shigella or hiv or aids or] OR [Title: "human immunodeficiency virus"] OR [Title: or] OR [Title: "acquired immunodeficiency syndrome"] OR [Title: or] OR [Title: "acquired immuno-deficiency

syndrome"] OR [Title: or tuberculosis or tb or malaria* or measles or] OR [Title: "whooping cough"] OR [Title: or pertussis or pertusses or hepatitis or meningitis] OR [Title: ncov* or 2019ncov or 19ncov or covid19* or covid or "sars-cov-2" or "sarscov-2" or "sars-cov2" or sarscov2 or] OR [Title: "sars coronavirus 2"] OR [Title: or] OR [Title: "severe acute respiratory syndrome coronavirus 2"] OR [Title: or] OR [Title: "severe acute respiratory syndrome corona virus 2"]] AND [Publication Date: (01/01/2000 TO 31/12/2022)]

Limited to Journals

82 records in Total

Inspec (Engineering Village/Elsevier)

3,556 records found in Inspec for 2000-2023: (((("artificial intelligence" OR "Machine learning" OR "neural network" OR "deep learning" OR "supervised learning" OR "unsupervised learning" OR "deep architecture" OR "computational intelligence" OR "computer reasoning" OR "machine intelligence" OR "support vector machine" OR "support vector network" OR "natural language processing" OR "data driven algorithm" OR perceptron OR "random forest" OR "ensemble learning" OR "reinforcement learning")) WN KY) AND (((LRTI or "lower respiratory tract infection" or influenza or flu or pneumonia or Bronchitis or bronchiolitis or diarrhea* or diarrhoea* or "viral gastroenteritis" or Campylobacter or salmonella or dysentery or "Escherichia coli" or "e coli" or "C difficile" or "Clostridium difficile" or norovirus* or "norwalk virus" or "infectious colitis" or "bacterial colitis" or "CMV colitis" or "Cytomegalovirus colitis" or "amoebic colitis" or "viral colitis" or Shigellosis or shigella or HIV or AIDS or "human immunodeficiency virus" or "acquired immunodeficiency syndrome" or "acquired immuno-deficiency syndrome" or Tuberculosis or TB or malaria* or measles or "whooping cough" or pertussis or pertusses or hepatitis or Meningitis OR nCoV* or 2019nCoV or 19nCoV or COVID19* or COVID or "SARS-COV-2" or "SARSCOV-2" or "SARS-COV2" or SARSCOV2 or "SARS coronavirus 2" or "Severe Acute Respiratory Syndrome Coronavirus 2" or "Severe Acute Respiratory Syndrome Corona Virus 2")) WN KY)) AND (JA WN DT) - {handicapped aids} WN CV
